# Nitric oxide nano-reactor DNMF/PLGA enables tumor vascular microenvironment and chemo-hyperthermia synergetic therapy

**DOI:** 10.1186/s12951-024-02366-y

**Published:** 2024-03-13

**Authors:** Ruoyao Wang, Long Cheng, Lingyun He, Chier Du, Haiyang Wang, Bohao Peng, Xiaoqing Yu, Weiwei Liu, Wenpei Luo, Haitao Ran, Lu Yang

**Affiliations:** 1 Department of Breast and Thyroid Surgery, The Second Affiliated Hospital of Chongqing Medical University, Chongqing, 400010 People’s Republic of China; 2 Department of Ultrasound, The Second Affiliated Hospital of Chongqing Medical University, Chongqing, 400010 People’s Republic of China

**Keywords:** Nitric oxide, Nanoparticle, Vessel normalization, Multi-modality image, Photo-thermal therapy

## Abstract

**Background:**

Breast cancer ranks first among malignant tumors, of which triple-negative breast cancer (TNBC) is characterized by its highly invasive behavior and the worst prognosis. Timely diagnosis and precise treatment of TNBC are substantially challenging. Abnormal tumor vessels play a crucial role in TNBC progression and treatment. Nitric oxide (NO) regulates angiogenesis and maintains vascular homeostasis, while effective NO delivery can normalize the tumor vasculature. Accordingly, we have proposed here a tumor vascular microenvironment remodeling strategy based on NO-induced vessel normalization and extracellular matrix collagen degradation with multimodality imaging-guided nanoparticles against TNBC called DNMF/PLGA.

**Results:**

Nanoparticles were synthesized using a chemotherapeutic agent doxorubicin (DOX), a NO donor L-arginine (L-Arg), ultrasmall spinel ferrites (MnFe_2_O_4_), and a poly (lactic-co-glycolic acid) (PLGA) shell. Nanoparticle distribution in the tumor was accurately monitored in real-time through highly enhanced magnetic resonance imaging and photoacoustic imaging. Near-infrared irradiation of tumor cells revealed that MnFe_2_O_4_ catalyzes the production of a large amount of reactive oxygen species (ROS) from H_2_O_2_, resulting in a cascade catalysis of L-Arg to trigger NO production in the presence of ROS. In addition, DOX activates niacinamide adenine dinucleotide phosphate oxidase to generate and supply H_2_O_2_. The generated NO improves the vascular endothelial cell integrity and pericellular contractility to promote vessel normalization and induces the activation of endogenous matrix metalloproteinases (mainly MMP-1 and MMP-2) so as to promote extravascular collagen degradation, thereby providing an auxiliary mechanism for efficient nanoparticle delivery and DOX penetration. Moreover, the chemotherapeutic effect of DOX and the photothermal effect of MnFe_2_O_4_ served as a chemo-hyperthermia synergistic therapy against TNBC.

**Conclusion:**

The two therapeutic mechanisms, along with an auxiliary mechanism, were perfectly combined to enhance the therapeutic effects. Briefly, multimodality image-guided nanoparticles provide a reliable strategy for the potential application in the fight against TNBC.

**Graphical Abstract:**

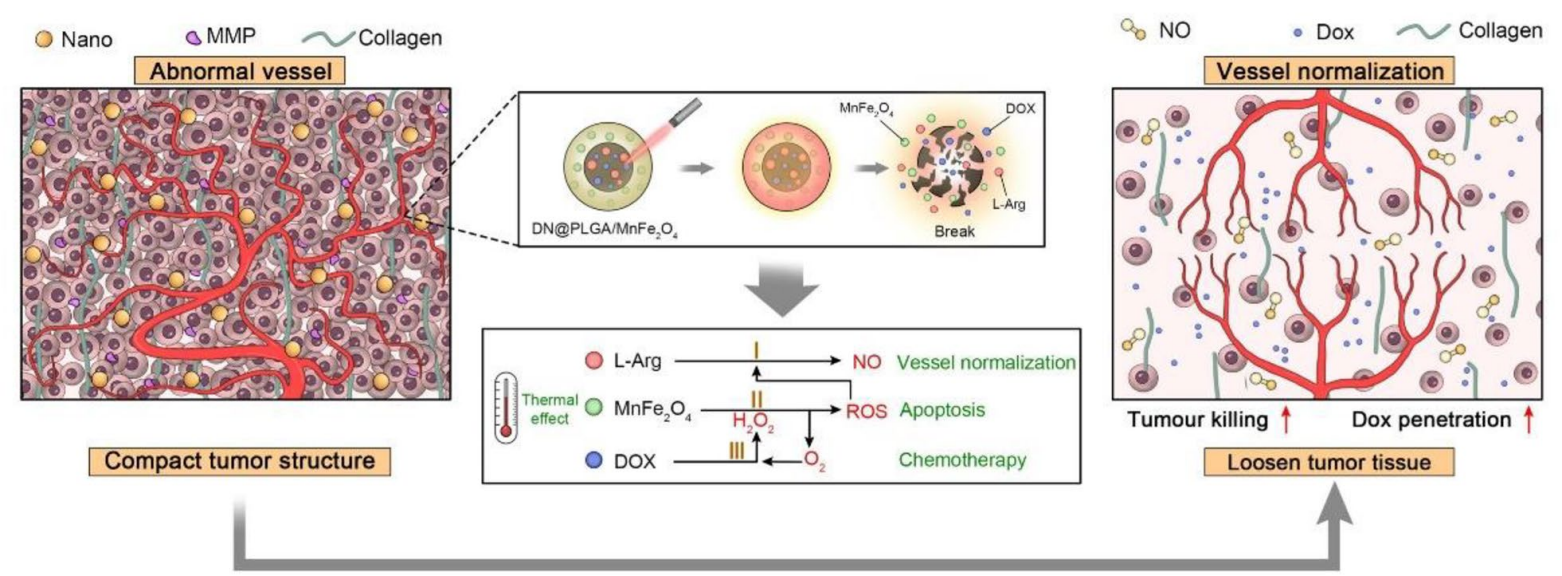

**Supplementary Information:**

The online version contains supplementary material available at 10.1186/s12951-024-02366-y.

## Introduction

In 2023, the incidence of breast cancer ranks first among all malignant tumors in female [1]. It poses a serious threat to female health, and triple-negative breast cancer (TNBC) is a subtype with a high invasion capability and the worst prognosis [2]. The lack of effective therapeutic targets and diagnostic methods is a vital reason for this problem [3]. Surprisingly, antibody drug conjugates show certain effect in advanced TNBC, the overall survival was improved for about half a year in previous clinical study [4]. However, with the continuous advancement of antitumor drugs, chemotherapy remains the most crucial treatment for TNBC. Anthracycline drugs such as doxorubicin (DOX) are the foundation for breast cancer chemotherapy [5]. Although studies investigating de-anthracyclines have shown an equal short-term effect in the neoadjuvant stage, the long-term result remains unclear [6]. Hence, several scholars assert the need to look at the use of anthracyclines from a fresh perspective, despite its certain cardiac toxicity [7]. In addition, early identification and therapeutic monitoring are crucial for patient prognosis, which requires suitable imaging methods and techniques that offer accurate imaging and real-time monitoring [8]. Proposing a novel theragnostic strategy for TNBC with optimized combination therapy and multimodal imaging would therefore be meaningful.

TNBC is characterized by its significantly higher microvascular density and increased expression of the vascular endothelial growth factor (VEGF), which leads to an abnormal microenvironment related to tumor angiogenesis. Angiogenesis with incomplete endothelial TNBC tumors causes drug leakage and inadequate delivery to tumor cells [9]. Moreover, a significant increase in collagen fibers surrounding microvessels was noted in the TNBC microenvironment, which resulted in a high intravascular pressure [10]. This hemodynamic change further prevents drug delivery during treatment. Vascular normalization includes a normal vascular endothelium, vascular density, and intravascular pressure, thereby serving as an effective adjuvant strategy for TNBC therapy.

Stable tumor vascular normalization relies on precise regulation of the dosage and the duration of angiogenesis and regulatory agents. Nitric oxide (NO), as a significant molecule, has profound effects on tumor cell proliferation, angiogenesis, and metastasis [11]. NO plays a crucial role in vascular normalization by improving endothelial cell integrity and pericellular contractility as well as enhancing oxygen perfusion at the tumor sites, which are beneficial for drug delivery and penetration [12]. On the other hand, the depredation of tumor extravascular collagen for reducing intravascular pressure is crucial, as it also benefits drug delivery. Matrix metalloproteinases (MMPs) can degrade the protein composition in the extracellular matrix (ECM), especially the matrix collagen [13]. They are regulated by tissue inhibitors of metalloproteinases (TIMPs). Past studies have proven that NO-induced activation of MMPs, including MMP-1 and MMP-2, can functionally destroy the tumor matrix collagen in promoting the delivery of antitumor drugs [14]. The selection of an appropriate NO donor in NO gas therapy is pivotal [15]. The biocompatible L-arginine (L-Arg) emerges as a potential NO donor that can spontaneously produce NO and L-citrulline through catalysis mediated by nitric oxide synthase (NOS) and reactive oxygen species (ROS) [16].

ROS can catalyze NO production and kill tumor cells. According to some studies, ROS has a certain therapeutic effect on TNBC [17]. Laser's *in-situ* irradiation of light-absorbing materials within tumors serves as a crucial means of producing exogenous ROS because laser radiation is capable of inducing a photothermal effect [18]. Because of their relatively poor heat resistance, tumor cells can be effectively inhibited with the photothermal effect. Thus, laser irradiation is also a novel treatment against tumor cells [19]. Hence, a good photothermal conversion material must be identified to generate the photothermal effect. Metal spinel ferrite nanoparticles (NPs), especially MnFe_2_O_4_ (Mn = Mn^2+^, Fe^2+^, and Co^2+^), have excellent light absorption and photothermal conversion abilities [20]. MnFe_2_O_4_ is a composite oxide NP with exceptional chemical stability and controllable size and shape. Moreover, spinel ferrite-loaded nanoplatforms have a good ability to enhance magnetic resonance imaging (MRI) [21], photoacoustic imaging (PAI), and T2 imaging. Ultrasmall spinel ferrites (UMFs) have an excellent ability to enhance T1 imaging, which is beneficial for the MRI diagnosis of breast cancer.

MRI is a commonly used and the most accurate single-examination technology for breast cancer, but it may be limited because of its non-real-time imaging, high cost, long inspection time, and even loud noise [8]. PAI, as a new high-resolution medical imaging technology, can provide more morphological and functional information [22]. However, its penetration depth is limited to some extent. No imaging technology is perfect, but multimodal imaging can compensate for the deficiencies of a single technology and share their advantages. Accordingly, the combination of MRI and PAI offers great potential for early diagnosis and treatment monitoring in breast cancer. NPs with observable navigation characteristics have gradually become research hotspots in the field of tumor therapy, such as efficient cargo loading and image control of cargo transportation, the NPs will help controllable navigation in tumor therapy [23–25].

**Scheme 1:**
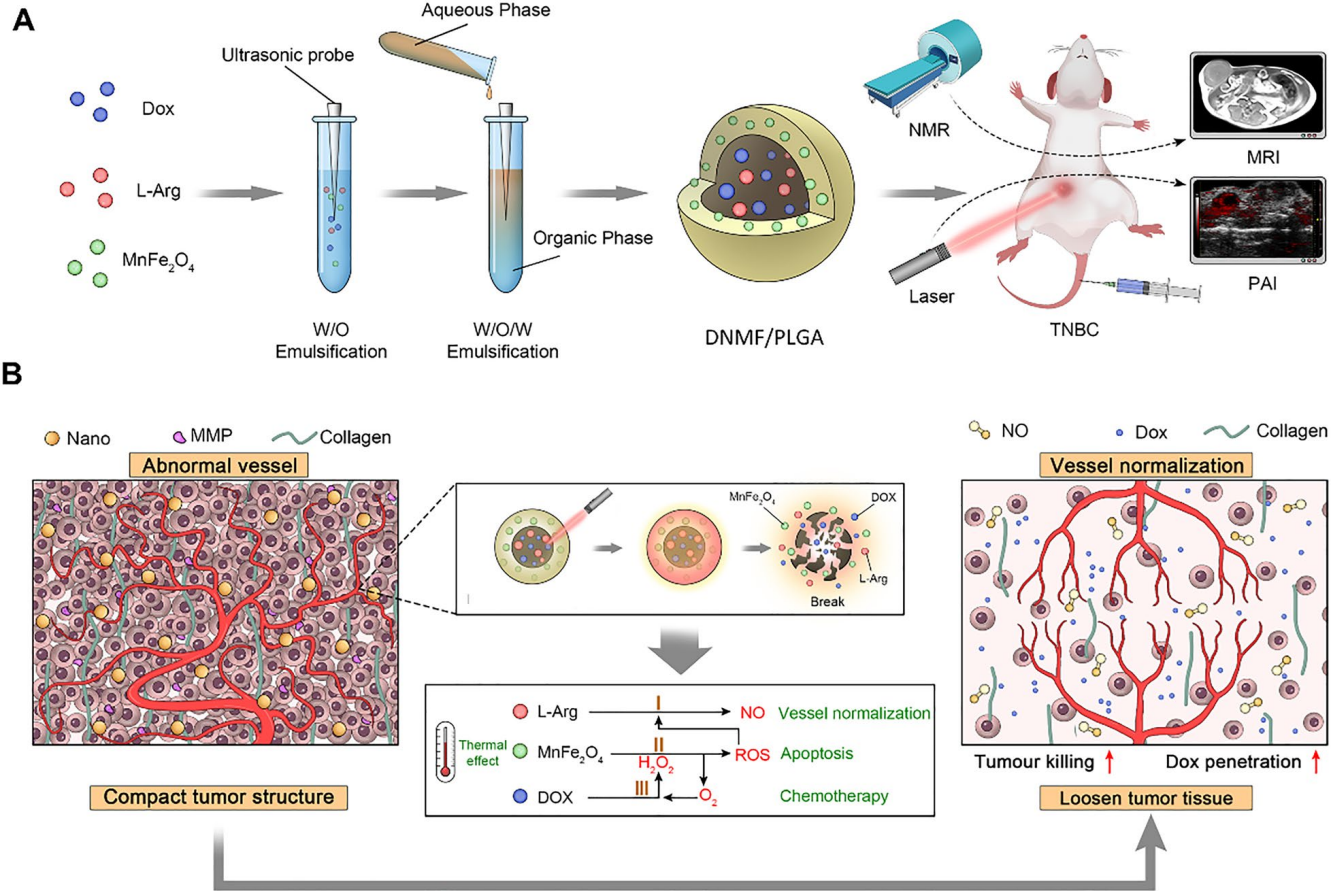
**A** Schematic illustration of the synthetic procedure of DNMF/PLGA NPs and the dual-modality of MRI/PAI. **B** The NO-induced vascular microenvironment remodeling and the synergistic chemo-hyperthermia as a composite strategy against triple-negative breast cancer (TNBC)

We prepared a nanoplatform with MnFe_2_O_4_, DOX, and L-Arg as the core, and poly (lactic-co-glycolic acid) (PLGA) as the shell. The unique core involving a combination of MnFe_2_O_4_, DOX, and L-Arg yields intriguing synergistic effects. After near-infrared (NIR) irradiation, MnFe_2_O_4_ catalyzes ROS production in the tumor cells, which, in turn, triggers the cascade catalysis of L-Arg to produce NO in the presence of ROS. In addition, DOX activates niacinamide adenine dinucleotide phosphate oxidase to generate and supply H_2_O_2_ for ROS production [26]. Consequently, low NO doses can normalize tumor blood vessels, reduce collagen in the extracellular matrix, and promote NP delivery and DOX penetration. Moreover, the chemotherapeutic effect of DOX, the photothermal effect of MnFe_2_O_4_, and even the ROS effect all together cause damage to TNBC cells, thereby resolving the problem of insufficient treatment (Scheme 1). Simultaneously, its ability to enhance MRI and PAI is beneficial for the diagnosis and therapeutic monitoring of TNBC. In addition, nano-load drug will result in superior detoxification systems that are more suitable for clinical practice [27–29]. Combining treatments with different mechanisms that allow accurate real-time monitoring will be a novel and potential theragnostic strategy for TNBC.

## Materials and methods

### Materials

The materials used in this study included carboxyl-modified PEGylated poly (lactic-co-glycolic acid) (lactide: glycolide = 50:50, PLGA = 25,000 Da MW, PEG = 5000 Da MW; PLGA-PEG5000-COOH) and oleic acid-coated manganese ferrite (MnFe_2_O_4_) nanoparticles (NPs, particle size = 3 nm, concentration = 8 mg/mL), which were obtained from Ruixi Biotechnology (Xi’an, China). Doxorubicin (DOX) was purchased from Beyotime Biotechnology (Shanghai, China). L-arginine (L-Arg), 2,7-dichlorodihydrofluorescein diacetate (DCFH-DA), poly (vinyl alcohol) (PVA; MW = 25,000), 1,1′-dioctadecyl-3,3,3′,3′-tetramethylindotricarbocyanine iodide (DiR), and 2-(4-amidinophenyl)-6-indolecarbamidine dihydrochloride (DAPI) were obtained from Sigma-Aldrich (Missouri, USA). All reagents used in this study were of analytical grade.

*Synthesis of DNMF/PLGA NPs:* PLGA-PEG (50 mg), MnFe_2_O_4_ (360 µL), DOX (1 mg), and L-Arg (1.25 mg/μL, 40 µL) were synthesized using a simple double-emulsion method, as per a previously described protocol [30]. In addition, DiR-labeled NPs and NPs without L-Arg, DOX, or MnFe_2_O_4_ were prepared by using the same method.

### Characterization of DNMF/PLGA NPs

The structure and morphology of the NPs were characterized using transmission electron microscopy (Hitachi H-7600, Tokyo, Japan). The average zeta potential and size were measured by the ZetaSizer series (Nano ZS90, Malvern Instrument, UK). The distribution of MnFe_2_O_4_, high-resolution images, elemental mapping images, and energy-dispersive spectroscopy (EDS) line-scan measurements were obtained using TEM (JEM 2100, JEOL, Tokyo, Japan). The light absorption of NPs was measured using UV–Vis-NIR absorption spectra (UV-3101PC, Shimadzu, Kyoto, Japan). High-performance liquid chromatography (HPLC) analysis was performed using an Onan LC-2010A HT system (Shimadzu, Kyoto, Japan). Fourier-transformed infrared spectroscopy (#6700, Nicolet, America) was also used. Confocal laser scanning microscopy (CLSM) images were acquired with an FV1000 system (Olympus, Tokyo, Japan). Cell apoptosis and cellular endocytosis of NPs were assessed by flow cytometry (FCM; BD LSRFortessa, Becton, Dickinson, USA). Photothermal hyperthermia performance was evaluated with 808-nm multi-mode pump laser irradiation (Shanghai Connet Fiber Optics, Shanghai, China). Photoacoustic (PA) images were obtained using the Vevo LAZR PA system (VisualSonics, Canada). Magnetization hysteresis loops of DNMF/PLGA NPs were measured using a vibrating sample magnetometer (PPMS-9, MicroSense, USA).

### In Vitro photothermal performance of DNMF/PLGA NPs

A DNMF/PLGA NPs solution was subjected to 808-nm laser irradiation at a power density of 1.5 W cm^−2^ using different MnFe_2_O_4_ concentrations (0, 0.25, 0.5, 1, 1.5, 2 mg/mL). In addition, a DNMF/PLGA NPs solution (2 mg/mL) was exposed to an 808-nm laser at different power intensities (0, 0.5, 1, 1.5, and 2 W cm^−2^). The resulting temperature changes in the DNMF/PLGA NPs upon irradiation with an 808 nm laser were recorded by using an infrared camera.

### DOX and NO release in vitro

For the DOX-release assay, 5 mg of DNMF/PLGA NPs were dispersed in PBS (1 mL) and placed in independent dialysis bags (4–8 kDa). These bags were immersed in flasks containing 100 mL of simulated body fluid at 37 °C with a rotation speed of 120 rpm. Prior to laser irradiation (808 nm, 1.5 W cm^−2^ for 5 min), the bags containing DNMF/PLGA NPs were shaken. The bags were then shaken for an additional 2 days. At different time points (0, 0.5, 1, 2, 4, 6, 12, 24, and 48 h), 1 mL of aliquots were collected from the three groups, and 1 mL of simulated body fluid was replenished in the flasks. The amount of DOX released in the supernatant was quantified by using a fluorescence assay at an excitation wavelength of 480 nm and an emission wavelength of 590 nm. The generation of NO from DNMF/PLGA NPs was measured using the Griess reagent, both without and with NIR laser irradiation (808 nm, 1.5 W cm^−2^, 5 min). To quantify the concentration of NO released at each time point, a standard curve was established using commercial NaNO_2_ at concentrations of 1, 3, 5, 7, and 10 µM. After laser irradiation, 60 µL of the supernatant was rapidly mixed with the Griess reagent and analyzed by using a UV–vis spectrophotometer at 540 nm.

### Cell culture and tumor-bearing mice model establishment

The murine 4T1 breast cancer cells were cultured in RPMI-1640 medium supplemented with 10% fetal bovine serum, 100 U/mL penicillin, and streptomycin under 5% CO_2_ at 37 °C. All female balb/c mice (weight: 16–20 g, age: 4–6 weeks) were obtained from Ensiweier Biotechnology (Chongqing, China) and housed in a humid environment with free access to food and water. All animal experimental procedures were conducted in accordance with the ethical standards of Chongqing Medical University. To establish 4T1 tumor-bearing mice, a PBS solution containing 1 × 10^6^ 4T1 cells (100 µL) was subcutaneously injected into the right mammary fat pad.

### NO release in 4T1 cells

The NO-specific probe DAF-FM DA was utilized to evaluate the release of NO in tumor cells. DAF-FM DA reacts with NO to produce a highly fluorescent compound called benzotriazole, which can be detected at an excitation wavelength of 495 nm and an emission wavelength of 515 nm. In a 24-well plate with coverslip glass, 50,000 4T1 cells were seeded in each well. After 24 h of incubation, the cells were inoculated in a fresh medium containing DAF-FM DA at a concentration of 10 µM for 1 h. Subsequently, the cells were divided into 6 groups, as follows: control, N/PLGA, NMF/PLGA, DNMF/PLGA, NMF/PLGA + laser, and DNMF/PLGA + laser. These groups were exposed to the respective treatments for 4 h. Following fixation with paraformaldehyde and staining with DAPI, fluorescence images reflecting the release of NO were captured by using an FV1000 microscope (Olympus, Tokyo, Japan).

#### In vitro biocompatibility assay

4T1 cells were seeded in a 96-well plate at the density of 1 × 10^4^ cells/well. The cells were then co-incubated with DNMF/PLGA NPs at different concentrations (0.05, 0.1, 0.2, 0.4, and 0.8 mg/mL) for 12, 24, and 48 h, respectively. Next, the cell viability was assessed by the CCK-8 assay, which is a standard method for measuring cell viability and proliferation.

### Synergistic chemo-hyperthermia therapy in vitro

4T1 cells were seeded in a 96-well plate at the density of 1 × 10^4^ cells/well. The cells were then divided into 6 groups: N/PLGA, DOX/PLGA, Laser only (1.5 W cm^−2^, 5 min), DNMF/PLGA, MF/PLGA + laser, DNMF/PLGA + laser (1.5 W cm^−2^, 5 min). The efficiency of synergistic chemo-hyperthermia was verified by the standard CCK-8 assay.

### Live-dead cell staining assay

4T1 tumor cells were seeded in 35-mm confocal dishes and subjected to different treatments: N/PLGA, DOX/PLGA, Laser only, DNMF/PLGA, MF/PLGA + laser, and DNMF/PLGA + laser. All laser irradiation was turned to 1.5 W cm^−2^ for 5 min. Then, the dishes were washed thrice with PBS, and 15 µL of Calcian-AM and 10 µL of PI were added to stain the cells for 20 min. Finally, the dishes were washed for another three rounds with PBS, followed by observation on the CLSM system.

### Apoptosis assay in vitro

4T1 cells were plated in a 6-well plate (4 × 10^5^ cells/well) and subjected to different treatments, as follows: N/PLGA, DOX/PLGA, Laser only, DNMF/PLGA, MF/PLGA + laser, DNMF/PLGA + laser, with the laser irradiation turned to 1.5 W cm^−2^ for 5 min. The cells were digested with trypsinization (Beyotime Biotechnology) and centrifuged at 1000 rpm for 5 min. The precipitates obtained were resuspended in PBS and analyzed by FCM after labeling with Annexin V-FITC and PI (co-incubation for 20 min).

### Intracellular endocytosis of DNMF/PLGA NPs

4T1 tumor cells were seeded into 35-mm confocal dishes and incubated with DNMF/PLGA NPs for 0, 0.5, 1, 2, and 4 h. The cell nucleus was stained with DAPI (500 µL, 10 min). The cells were washed thrice with PBS and then observed by CLSM. Furthermore, the 4T1 tumor cells were seeded into a 6-well plate (4 × 10^5^ cells/well) for 24 h. The DNMF/PLGA NPs were incubated with tumor cells for 0, 0.5, 1, 2, and 4 h. Next, the cells were digested and resuspended in PBS, followed by analyses by FCM.

### Intracellular ROS detection

The 4T1 tumor cells seeded in confocal culture dishes or microplates were randomly divided into five groups in accordance with different treatments, including N/PLGA, NMF/PLGA, DNMF/PLGA, NMF/PLGA + laser, and DNMF/PLGA + laser. After 4 h of co-incubation, the cells in both the laser group were irradiated at 1.5 W cm^−2^ for 5 min. After an additional 4 h of co-incubation, fluorescent DCFH-DA was used to detect the ROS levels in cells. All dishes were imaged by CLSM, and all cells were collected for FCM analysis.

### MnFe_2_O_4_ detected in vivo and in vitro

PLGA-PEG (50 mg), MnFe_2_O_4_ (20, 40, 80, 160 µL), DOX (1 mg), and L-Arg (1.25 mg/μL, 40 µL) were synthesized using a simple double-emulsion method, following a previously described protocol [30]. DNMF/PLGA nanoparticles concentration was quantified at 10 mg/mL, and the content of MnFe_2_O_4_ in nanoparticles was quantified by ICP-MS (NexlON300D, Perkin Elmer, America). The decomposition and release of Mn^2+^ and Fe^2+^ from MnFe_2_O_4_ under physiological conditions (PBS) with or without laser irradiation was also quantified by ICP-MS.

Mice bearing 4T1 tumor (-100 mm^3^) were intravenously injected and treated with the NPs (N/PLGA, NMF/PLGA, DNMF/PLGA, NMF/PLGA + laser, DNMF/PLGA + laser) at the concentration of 2 mg/mL. After 6 h of the injection, the mice were sacrificed and the tumors were weighed. Next, after homogenization at 4500 rpm for 1 min using Precellys 24 tumor homogenizer (Bertin Technologies, France), the cells were centrifuged at 12000 rpm for 15 min. Finally, the Fe content in nanoparticles was quantified by ICP-MS (NexlON300D, Perkin Elmer, America).

### Intracellular H_2_O_2_ detection

The cells seeded in confocal culture dishes were randomly distributed into five groups in accordance with treatments performed, as N/PLGA, NMF/PLGA, DNMF/PLGA, NMF/PLGA + laser, and DNMF/PLGA + laser groups. After co-incubation for 4 h, the cells in both the laser group were irradiated at 1.5 W cm^−2^ for 5 min. After an additional 4 h of co-incubation, the Hydrogen Peroxide Assay kit was used to detect the H_2_O_2_ levels in the tumor cells.

### Anti-tumor therapy in vivo

The experimental mice were randomly assigned to four groups of 5 each and treated with saline (control), Laser only, DNMF/PLGA, and DNMF/PLGA + laser, respectively. The concentrations of NPs were 5 mg/mL, and the laser irradiation was performed at 1.5 W cm^−2^ for 10 min. After treatments, the body weights and tumor size were recorded every 3 days. After the end of the observation period (14 days), the tumors were removed and the tumor weight was measured. Then, the tumors were fixed in a 4% formaldehyde solution and subjected to pathological hematoxylin and eosin (H&E) staining. The tumor histological changes were identified using T dT-mediated dUTP Nick-End Labeling (TUNEL), VEGF antibody (Service Bio, Wuhan, China), and Ki-67 antibody staining (n = 3). The vital organs (such as the heart, liver, spleen, lung, and kidney) were excised for H&E staining to evaluate the systemic toxicity. Image-Pro Plus 6.0 software (Media Cybernetics, MD) was employed for further analysis and scoring.

### In vivo biosafety of DNFM/PLGA NPs in Kunming mice

Female Kunming mice (n = 5) were intravenously injected with DNFM/PLGA NPs (2 mg/mL, 1 mL), while the mice in the control group (n = 5) did not receive any treatments. The blood and vital organs were collected at 0, 1, 7, and 14 days after injection for routine blood examination, serum biochemical analysis, and histological analysis using H&E staining.

### ONOO^−^ detection in tumor

Owing to the instability of ONOO^−^, 3-nitrotyrosine (3-NT), formed with nitration of tyrosine residues of proteins by ONOO^−^, is a common alternative representing the presence of ONOO^−^. Mice bearing 4T1 tumor (-100 mm^3^) were intravenously injected and treated with the NPs (N/PLGA, NMF/PLGA, DNMF/PLGA, NMF/PLGA + laser, DNMF/PLGA + laser) at the concentration of 2 mg/mL. After 48 h of injection, the mice were sacrificed and the tumors were fixed in 4% formaldehyde and processed for paraffin sectioning. 3-NT was stained using a mouse anti-3-nitrotyrosine antibody (Santa Cruz Biotechnology, Dallas, USA). Images of the tumor sections were captured by using an optical microscope, while the intensity of 3-NT was analyzed with Image-Pro Plus 6.0 software.

### Expression and activity of MMPs

Western blot was performed for matrix metalloproteinases (MMPs) examination. After 48 h of intravenous injection of the NPs and laser irradiation, the tumors were excised and immersed in RIPA buffer containing PMSF (Beyotime Biotechnology) and PhosSTOP Phosphatase Inhibitor Cocktail (Roche, Switzerland) for 30 min, and then homogenized at 4500 rpm for 1 min using Precellys 24 tumor homogenizer (Bertin Technologies, France), followed by centrifugation at 12000 rpm for 15 min. The total proteins were obtained through centrifugation, and 20 µg proteins were processed for Western blotting with specific rabbit anti-MMP-1 and MMP-2 antibodies (Abcam, Hong Kong). The activity of MMPs was evaluated through in situ zymography with the EnzChek Gelatinase/Collagenase Assay Kit (ThermoFisher Scientific, Shanghai, China).

### Collagen I assay

After 48 h of NPs injection and laser irradiation, the tumors were removed and frozen-sectioned for staining with rabbit anti-mouse collagen I antibody (1:200, Abcam, Hong Kong) and Alexa Flour 647 donkey anti-rabbit IgG (Life Technologies, China). Fluorescent images were obtained using CLSM (Ex 649 nm, Em 664 nm) and analyzed using Image-Pro Plus 6.0 software.

### Distribution of NO in the tumor area

After 6 h of an intravenous injection of the NPs, the tumor sites were irradiated using an 808 nm laser. After 2 h, DAF-FM DA (3 mg/kg) was intratumorally injected into the mice. After 30 min, the tumor tissues were harvested, embedded in OCT, frozen and sliced, and observed by CLSM. The tumor tissues were sectioned into 10-µm thickness, and images were representative sections from three mice per group.

### Assay of tumor vessels

After 48 h of NPs injection and laser irradiation, the tumors were removed and fixed in 4% paraformaldehyde for 24 h, paraffin-embedded, and sectioned into 10–20 µm thickness. Next, the sections were dewaxed in xylene and rehydrated in a graded series of alcohol. Antigen retrieval was conducted in citric acid buffer (pH 6.0) for 10 min at 98** °C**. The tumor sections were blocked in 2% normal goat serum (1:200; Protein Tech Group, Chicago, USA) for 1 h at room temperature. Then, the sections were incubated overnight at 4** °C** with an anti-cluster of differentiation (CD)-31 antibody (1:500; ab28364; Abcam, Cambridge, UK) and α-smooth muscle actin (SMA) antibody (1:100; 14,395-1-AP; Protein Tech Group, Chicago, USA). Next, these sections were washed and incubated with rhodamine-conjugated goat anti-rabbit IgG-FITC (1:200; sc-2359; Santa Cruz Biotechnology, Dallas, USA) for 40 min at room temperature. The nucleus was counterstained using DAPI for 15 min at room temperature (00-4959-52; Invitrogen; Thermo Fisher Scientific, Massachusetts, USA), and the tissues were visualized using a fluorescent microscope (× 20–× 1000; Leica DM6000B; Leica Microsystems GmbH, Wetzlar, Germany). For each section, a total of 5 images were taken from randomly selected fields of view.

### CircRNAs profiling analysis

Before image and base recognition, premier reads were harvested from illumina Novaseq 6000 sequencer. Cutadapt software was used to remove the connector and lower-quality reads, and only high-quality clean reads were retained. The edgeR software (v3.16.5) was employed to normalize the data and differentially expressed mRNAs were analyzed.

### Bioinformatics analysis

Gene Ontology (GO) and Kyoto Encyclopedia of Genes and Genomes (KEGG) analysis were conducted for the target differentially expressed mRNAs using DAVID (Database for Annotation, Visualization, and Integrated Discovery).

### In vitro fluorescence and in vivo biological distribution

The different concentrations of DiR-labeled NPs (0.25, 0.5, 1, 1.5, and 2 mg/mL) were detected by a live fluorescence imaging system (Fx7 Ir Spectra, Vilber Lourmat, France). Furthermore, the 4T1 cell-bearing-tumor mice were intravenously injected with DiR-labeled NPs (2 mg/mL, 1 mL). After anesthetizing with an intraperitoneal injection of 3% pentobarbital sodium (60 mg/mL, 0.15 mL), the live fluorescence imaging system was used to evaluate the biological distribution of NPs at different time points (0, 1, 3, 6, 12, and 24 h). The mice were sacrificed after 24 h of injection, and the major organs (such as the heart, liver, spleen, lung, and kidney) and the tumors were harvested for ex vivo fluorescence imaging.

### In vitro and in vivo MRI

MRI experiments were conducted using a clinical 3 T MR scanner (HDXT2012; GE Medical Systems, Fairfield, USA). Different concentrations of DNMF/PLGA (0, 0.625, 1.25, 2.5, 5, 10 mg/mL) were placed in 15-mL EP tubes for in vitro MRI under the following T1-imaging parameters: TR, 115 ms; TE, 9.21 ms; flip angle, 30°; FOV, 245 mm; matrix, 256 × 256; slice thickness, 1 mm. The MRI T1-signal intensities within the region of interest (ROI) were measured, and the corresponding relaxation rates were calculated. For the in vivo MRI, the model rats (n = 3) were anesthetized and intravenously injected with the DNMF/PLGA NPs solution (2 mg/mL). The T1 imaging parameters were set as follows: TR, 3200 ms; TE, 80 ms; flip angle, 30°; FOV, 245 mm; matrix, 256 × 256; slice thickness, 1 mm. Subsequently, transverse MRI images were collected at the nanoparticle’s concentration of 2 mg/mL.

### In vitro and in vivo PAI

For in vitro PAI of DNMF/PLGA NPs, the samples were scanned with an excitation wavelength range of 680–970 nm on a PAI system (Vevo LAZR, Canada) at a PA gain of 40 dB. The tunable laser parameters used for the PAI system were set as follows: type, flash lamp pumped Q-switched Nd: YAG laser with an optical parametric oscillator and second harmonic generator; frequency, 20 Hz; wavelength, 680–970 nm; step size, 2 nm; pulse duration, 4–6 ns; peak energy, 45 ± 5 mJ (at 20 Hz); spot size: 24 mm^2^ (1 × 24 mm). The PA images and the relative PA signal values of the DNMF/PLGA NPs at different concentrations (0.1, 0.2, 0.4, 0.6, and 0.8 mg/mL) were obtained at wavelengths of 680–690 nm. For in vivo PA imaging, at the tumor sizes of approximately 160 mm^3^, the tumor-bearing mice (n = 3) received an intravenous administration of DNMF/PLGA NPs (2 mg/mL, 1 mL). The PA signals in the tumor regions were recorded at determined time points after the injection.

### Statistical analysis

All quantitative data were expressed as the mean ± standard deviation (SD). Statistical analysis was performed using Graph-Pad 6.0 (La Jolla, CA, USA). Differences between the two groups were analyzed using Student’s unpaired or paired t-tests. Differences among multiple groups were calculated by one-way analysis of variance (ANOVA), and one-way or two-way ANOVA with repeated measures with Tukey’s or Dunnett’s post-hoc test. The statistical tests were two-sided and p < 0.05 was considered to indicate statistical significance. All experiments were performed using n ≥ 3 biological replicates.

## Results and discussion

### Fabrication and characterization of DNMF/PLGA NPs

**Fig. 1:**
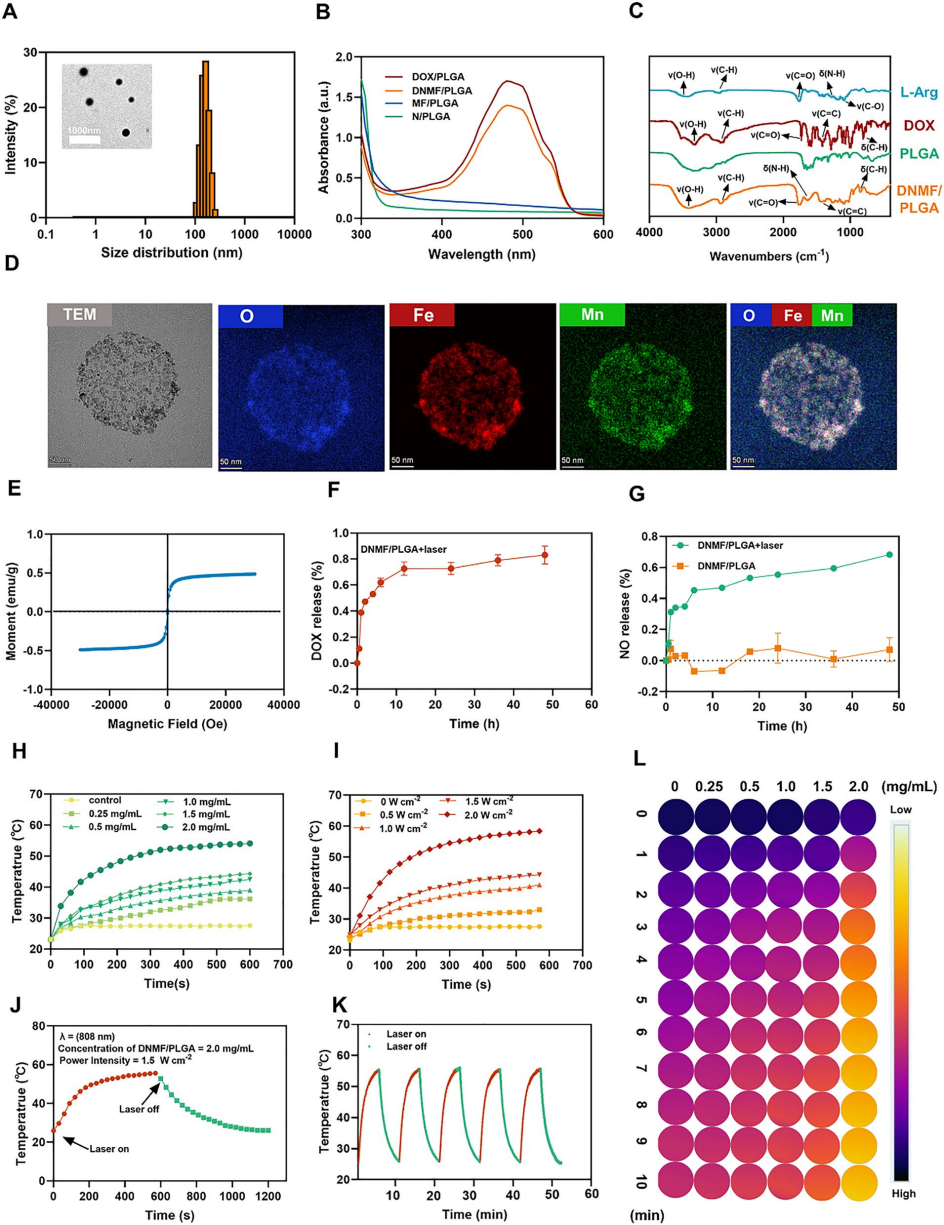
Characterization of DNMF/PLGA NPs. **A** The size distribution using DLS and the structure and morphology using TEM. **B** UV–vis absorbance spectra of the N/PLGA, DOX/PLGA, MF/PLGA, and DNMF/PLGA NPs. **C** The FTIR spectrum of different samples including L-Arg, DOX, PLGA, and DNMF/PLGA. **D** High-resolution TEM elemental mapping of Mn, Fe, and O, along with TEM elemental line-scan of the NPs. **E** Magnetization hysteresis loops of the nanoparticles at 300 K ranging from – 30 to + 30 kOe. **F** Cumulative DOX release in PBS (pH 6.8) within 48 h. **G** The NO-release status of both the treatment groups within 48 h, including DNMF/PLGA only and DNMF/PLGA + laser (1 W cm^−2^, 5 min). **H** Heating curves of various concentrations of DNMF/PLGA NPs upon 808-nm laser irradiation (1.5 W cm^−2^ for 10 min). **I** The temperature curves of DNMF/PLGA NPs (2.0 mg/mL) exposed to 808-nm laser at different power densities. **J** Photothermal conversion efficiency of DNMF/PLGA NPs exposed to 808-nm laser (1.5 W cm^−2^ for 5 min). **K** The photothermal stability of DNMF/PLGA NPs under five repeated heating/cooling cycles. **L** Infrared thermal images of DNMF/PLGA NPs at different concentrations under 808-nm laser (1.5 W cm.^−2^)

The synthetic NPs rationally loaded MnFe_2_O_4_, DOX, and NO donor L-Arg with PLGA shells (denoted as DNMF/PLGA NPs). DNMF/PLGA NPs were used for MRI/PAI dual imaging-guided and laser-triggered in situ DOX and NO release in passive and intracellular targeted photothermal therapy and DOX-induced chemotherapy. Specifically, Scheme 1 vividly illustrates the procedure of DNMF/PLGA NP synthesis. The transmission electron microscopy (TEM) image of the DNMF/PLGA NPs revealed a spherical morphology and uniform distribution in PBS (Figs. 1A, Additional file 1: Figure S14). The average size of the NPs and zeta potential were 236.2 ± 4.015 nm and 7.030 ± 0.514 mV, respectively (Figs. 1A, Additional file 1: Figure S1). Similarly, the sizes of the control NPs, including L-Arg NPs (N/PLGA), DOX NPs (DOX/PLGA), and MnFe_2_O_4_ NPs (MF/PLGA) were determined to be 178 ± 1.559, 191 ± 1.249, and 204 ± 2.707 nm (Additional file 1: Figure S3), respectively. We inferred that, with increased material types, the average size inevitably increased under the same experimental conditions. However, past studies have shown that NPs with an average size of approximately 200 nm have a good drug-loading ability and an EPR effect for retention within the tumor [31]. Importantly, the average size and zeta potential of DNMF/PLGA NPs were similar within 7 days, even under four different biological conditions (PBS, FBS, RPMI 1640 and saline), indicating the desirable stability and good dispersibility of NPs (Additional file 1: Figures S1, S2, S13).

The encapsulation efficiency and loading capacity increased from 39.03% to 58.3% and 0.37% to 2.62% with the addition of MnFe_2_O_4_, respectively. An initial MnFe_2_O_4_ input of 160 μL was selected for further experiments because it offers a good balance in the imaging capability, photothermal efficacy, and appropriate size of the NPs (Additional file 1: Figure S4A, B). Meanwhile, 19.9 ± 2.2 μg/mg L-Arg and 31.7 ± 2.6 μg/mg DOX were loaded in the DNMF/PLGA NPs, and the encapsulation of L-Arg and DOX were 62.4 ± 2.6 and 74.0 ± 2.6 μg/mg, respectively, which can release sufficient NO and DOX for tumor therapy (Additional file 1: Figure S15).

Concurrently, elemental line-scan mapping revealed that Mn, Fe, and O were present in the NPs (Fig. 1D). The detection of the paramagnetic properties of the NPs indicates their potential as an excellent MRI contrast agent (Fig. 1E), which further indicates the successful loading of MnFe_2_O_4_. While the metal decomposition and release from MnFe_2_O_4_ under physiological conditions was also investigated. In brief, the release of Mn^2+^ was more than Fe^2+^, the accumulated release of Mn^2+^ and Fe^2+^ increased over time, and the release increased after laser irradiation (Additional file 1: Figure S16A, B), which is consistent with the previous study [32, 33]. N/PLGA and MF/PLGA showed no special absorption peak at 300–600 nm, while DOX/PLGA and DNMF/PLGA showed the same absorption peak at 480 nm, which further confirmed the successful loading of DOX (Fig. 1B). Fourier-transformed infrared spectroscopy of DOX, PLGA, and L-Arg had a similar wave. DNMF/PLGA NPs also confirmed the loading of L-Arg and DOX (Fig. 1C). Following 808-nm laser irradiation for 5 min, fast DOX release was observed before 12 h, and a little release was noted from 12 to 48 h, exhibiting the laser as a trigger for drug release (Fig. 1F).

### Photothermal performance of MnFe_2_O_4_-based NPs

Various concentrations of DNMF/PLGA NP solutions (0.25, 0.5, 1.0 1.5, and 2.0 mg/mL) were exposed to 808-nm laser (1.5 W/cm^2^, 10 min) (Fig. 1H). The temperature increased with an increase in the concentration and irradiation time at the same laser intensity. Figure 1L also exhibited the same trend with images. The DNMF/PLGA NP solution (2 mg/mL) was irradiated with an 808 nm laser at varying power intensities (0.5, 1.0, 1.5, and 2.0 W/cm^2^) (Fig. 1I). The temperature variations were monitored through infrared thermal imaging. The temperature also increased with an increase in the power intensity and irradiation time at the same concentration of NPs. To further test the temperature sensitivity, the NP solution (2 mg/mL) was exposed to the 808-nm laser. The temperature of the solution quickly increased from room temperature to 60 °C within 600 s and then reduced to room temperature for approximately 600 s after the laser was turned off (Fig. 1J). Furthermore, 5 cycles of repeated heating/cooling attained the same top temperature within the same period (Fig. 1K**)**, indicating good photothermal stability of the DNMF/PLGA NPs.

### NIR laser-triggered NO generation

**Fig. 2:**
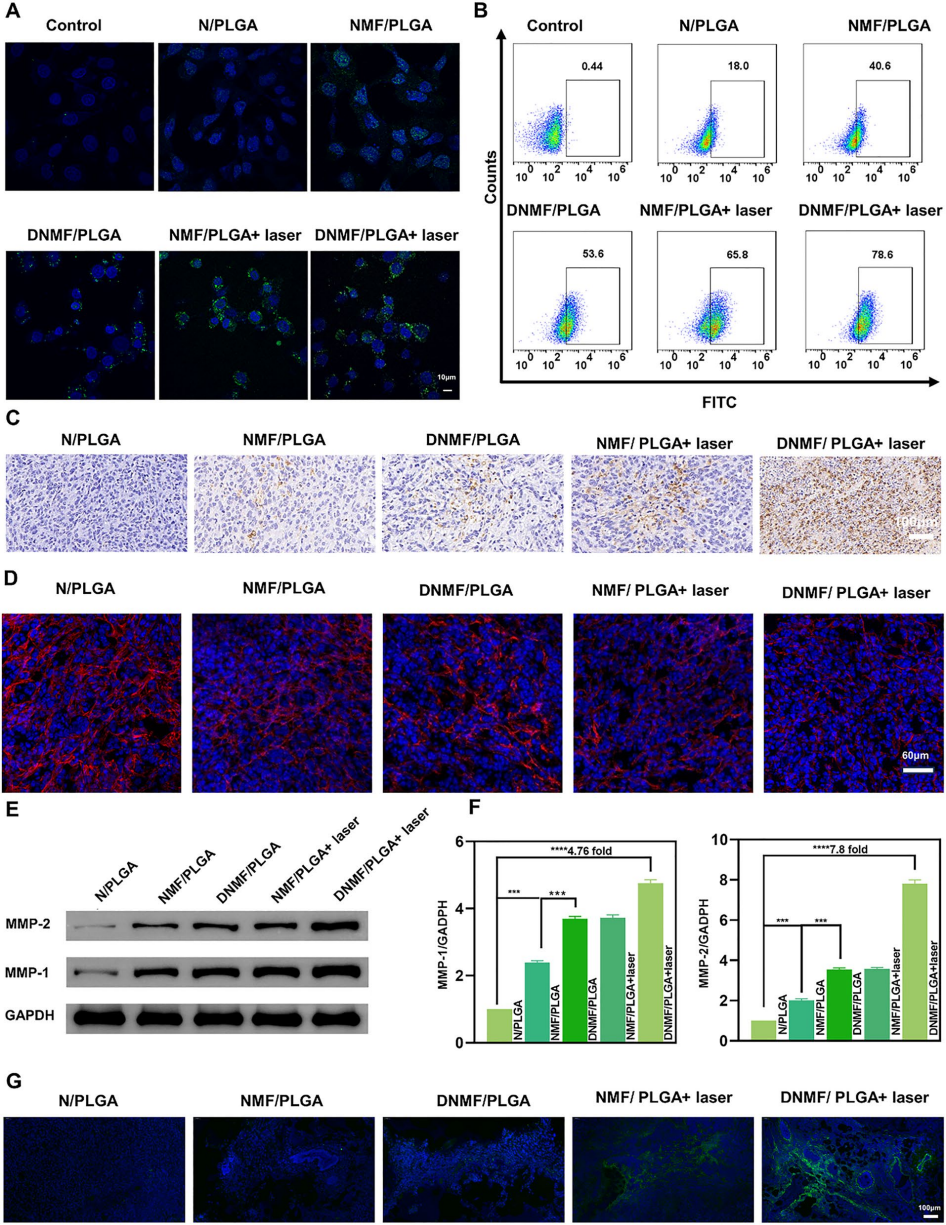
**A** After different treatments, the NO level in 4T1 cells was detected using a DAF-FM DA fluorescent probe by CLSM. **B** The NO level in 4T1 cells was detected using FCM. **C** Immunohistochemical examination of 3-NT (brown) in the tumor tissues. **D** Collagen I immunofluorescent staining of tumors (Red, collagen I; Blue, DAPI). **E** The increased expressions of MMP-1 and MMP-2 were detected by Western blotting. **F** Quantitative analysis of MMP-1 and MMP-2 variations. **G** After different treatments for 6 h, the NO level in vivo was detected using immunofluorescent staining (blue, cells; green, NO). The experiments were repeated thrice independently. ANOVA with Tukey’s post-hoc test. *p < 0.05, **p < 0.01, and ***p < 0.001

Previous study showed different ways of NO production, and the NO donor were also different, mainly including L-Arg and Nitroso compounds [34–36] (Additional file 1: Table S1). NIR-irradiated DNMF/PLGA NPs offer a smart and controllable method to simultaneously achieve ROS-responsive NO release. NO generation unveiled the NIR laser-triggered dependence of DNMF/PLGA NPs [37]. As shown in Fig. 1G, in NPs without or with laser irradiation, NO generation displayed different statuses. With laser irradiation, continuous NO generation lasted for 48 h, while a small amount of NO was generated without laser irradiation even for 48 h. NO was inferred to be generated from the released L-Arg because of the photothermal effect of ROS. When compared with the sudden release of other NO donors such as diazeniumdiolate, the NPs appeared to be continuously released under a laser trigger, which might be more suitable for tumor vessel normalization [38]. NO generation within the 4T1 cells was marked using the DAF-FM DA fluorescence probe in six groups and visualized using CLSM and FCM (Fig. 2A, B). NO (green fluorescence) was found to aggregate around cell nuclei (blue fluorescence) in the four groups (NMF/PLGA, DNMF/PLGA, NMF/PLGA + laser, and DNMF/PLGA + laser), while the DNMF/PLGA + laser group exhibited the strongest green fluorescence, indicating the highest NO generation for its laser trigger, as well as L-Arg and DOX release. FCM quantitatively analyzed NO generation in the 4T1 cells, which further confirmed the CLSM result (Additional file 1: Figure S7A, B). Under the same grouping, the NO-specific fluorescence probe was used to assess the penetration of NO released at the tumor sites. The release of NO in the tumor sites showed the same photothermal response as the cell groups (Figs. 2G, Additional file 1: Figure S7C). The photothermal effect of MnFe_2_O_4_ and DOX release promoted ROS production for more NO generation. To learn more about ROS, dichlorodihydrofluoresc ein (DCF) fluorescence was employed to characterize ROS generation using CLSM and FCM. The DNMF/PLGA + laser group exhibited the strongest green fluorescence, indicating that most ROS were generated in this group, which further confirms our inference (Additional file 1: Figure S6A). The corresponding results of FCM concurred with these results (Additional file 1: Figure S6B, C).

NO exerts two crucial effects in tumor therapy, namely vascular dilation for drug delivery and ONOO^−^ formation for MMP activation [39]. The active MMP enzymes can hydrolyze the collagen around tumor vessels to reduce intravascular resistance. Vascular dilation and the reduction of intravascular resistance are beneficial for drug delivery and penetration (Scheme 1B). NO reacts with the superoxide anion (O2^•−^, ROS production) in tumor cells to form ONOO^−^. Because the direct determination of ONOO^−^ in vitro is difficult, 3-nitrotyrosine (3-NT) is commonly used as a biomarker for ONOO^−^ activity determination in vivo [40]. Immunohistochemical staining of TNBC tumor tissues against 3-NT were performed at 48 h after NPs were injected intravenously. The 3-NT expression in the five groups (i.e., N/PLGA, NMF/PLGA, DNMF/PLGA, NMF/PLGA + laser, and DNMF/PLGA + laser) were different. The N/PLGA group exhibited no 3-NT expression, whereas the DNMF/PLGA + laser group exhibited the strongest expression because most ONOO^−^ was produced in this group (Fig. 2C). The results of the quantitative analysis of 3-NT expression confirmed these results (Additional file 1: Figure S10A). Past studies have shown that increased ONOO^−^ activity may lead to an increased expression of MMP enzymes. The most representative MMP enzymes are MMP-1 and MMP-2. Tumor ECM degradation is usually induced by the overexpression of MMPs and other proteases [41]. After the cells were treated with the five NPs (i.e., N/PLGA, NMF/PLGA, DNMF/PLGA, NMF/PLGA + laser, and DNMF/PLGA + laser), MMP-1 and MMP-2 in TNBC tumors were detected by western blotting. The MMP-1 and MMP-2 levels in the DNMF/PLGA + laser group significantly increased when compared with those in the N/PLGA group (Fig. 2E). The quantitative analysis exhibited a 4.76- and 7.8-fold increase, respectively (Fig. 2F). NPs with DOX, L-Arg, MnFe_2_O_4_, and laser irradiation displayed a better ability to generate NO. MMP activation both in vitro and in vivo further confirmed our inference that MnFe_2_O_4_ can catalyze H_2_O_2_ production in tumor cells to produce ROS after irradiation, L-Arg can produce NO in the presence of ROS, and DOX activates niacinamide adenine dinucleotide phosphate oxidase to produce and supply H_2_O_2_.

Collagen I is the main ECM component in solid tumors and is believed to be a crucial barrier against drug penetration in various cancers [42]. Increased MMP levels contributed to the reduction in collagen I levels. Collagen I levels were also determined in the TNBC tumors treated with the five NPs [43]. When compared with the N/PLGA group, the collagen I levels were significantly reduced in the DNMF/PLGA + laser group (Fig. 2D). Following the quantitative analysis, the reduction was found to be 1.85-fold, indicating good collagen I degradation (Additional file 1: Figure S10B).

### In vitro endocytosis and synergistic therapy of DNMF/PLGA NPs

**Fig. 3:**
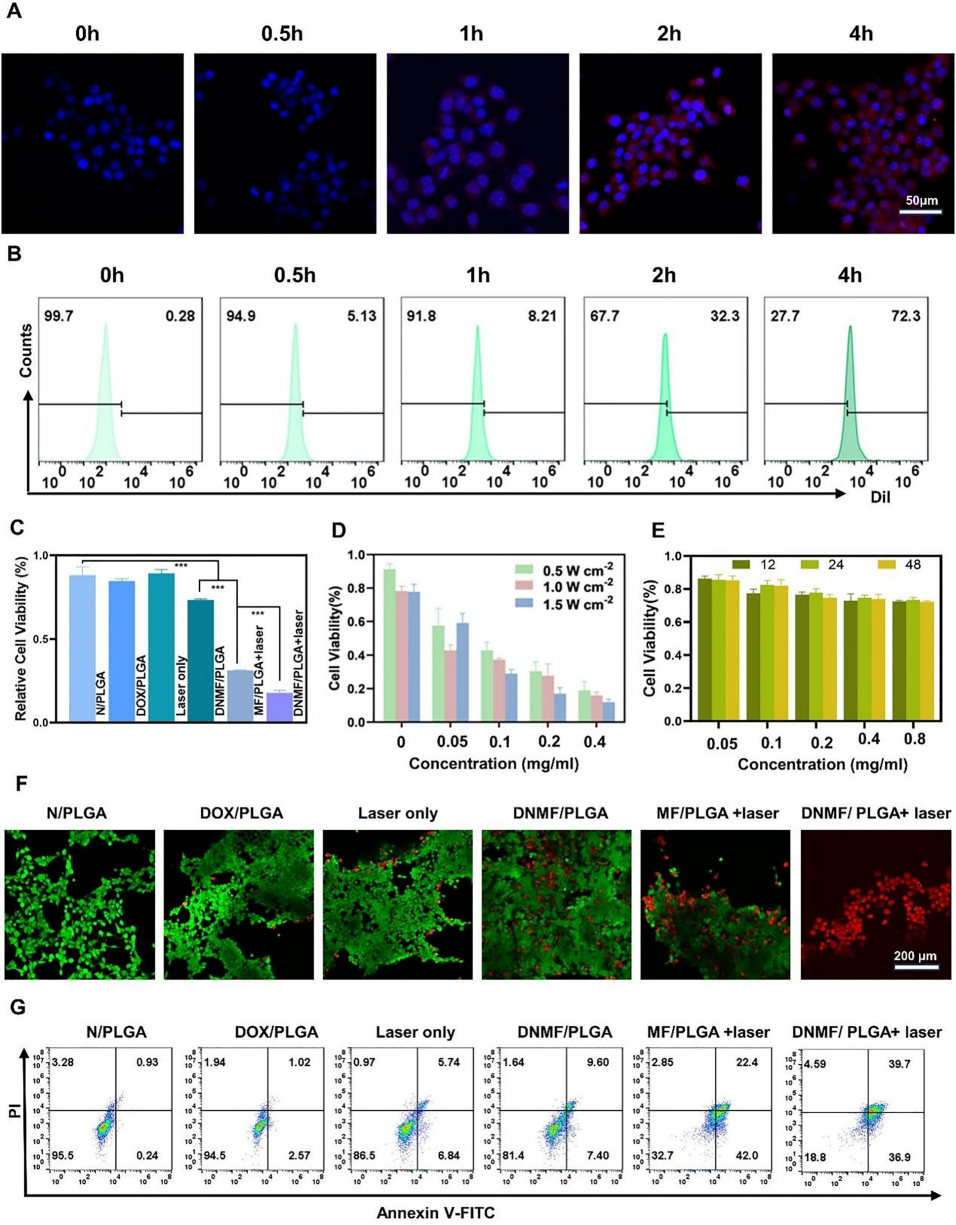
In vitro endocytosis and synergistic therapy of DNMF/PLGA NPs. **A** After 4T1 cells were incubated for 0, 0.5, 1, 2, and 4 h, the intracellular uptake of nanoparticles was observed using CLSM and **B** FCM, Red, DOX; Blue, DAPI. **C** Relative cell viability after different treatments, including N/PLGA, DOX/PLGA, Laser-only, DNMF/PLGA, MF/PLGA + laser, and DNMF/PLGA + laser. **D** The relative cell viabilities of DNMF/PLGA NPs at different concentrations (0, 0.05, 0.1, 0.2, and 0.4 mg/mL) under 808-nm laser irradiation at different power intensities of 0.5, 1, and 1.5 W cm^−2^ for 5 min. **E** Relative cell viabilities of 4T1 cells co-incubated with DNMF/PLGA NPs at different concentrations for different durations. **F** The CLSM images of 4T1 cells stained by PI and calcian-AM. **G** FCM apoptosis assay of 4T1 cells stained by Annexin-FITC and PI after different treatments. The power density was 1.5 W cm^−2^ and the irradiation time was 5 min

Endocytosis of DNMF/PLGA NPs (1 mg/mL) in 4T1 cells was detected through CLSM. After 0.5, 1, 2, and 4 h of co-incubation, the red fluorescence of DOX was around the blue fluorescence of the cell nucleus, while the red fluorescence intensity increased with an increase in the co-incubation time (0–4 h), which indicated the good endocytic effect and time dependency of the NPs by the 4T1 cells (Figs. 3A, Additional file 1: Figure S8). Furthermore, the endocytosis fluorescence intensity in the 4T1 cells was determined using FCM, which confirmed the results of CLSM (Fig. 3B).

The therapeutic effect in vitro was determined through the standard CCK-8 assay. The cells were divided into six groups, namely PLGA, DOX/PLGA, laser only, DNMF/PLGA, MF/PLGA + laser, and DNMF/PLGA + laser. In these figures, the DOX/PLGA group and the laser only group showed unsatisfactory treatment. After adding MnFe2O4 nanoparticles into the PLGA shell, the laser treatment was significantly improved. In order to achieve better therapeutic effect, we added L-arginine to produce NO gas. Reduce the effect of NO gas on tumor blood vessels in solid tumors was not considered in vitro, only the addition of NO gas still promoted tumor apoptosis to a certain extent. The DNMF/PLGA + laser group displayed the best therapeutic effect, which confirmed the inference of DOX release and photothermal effect after laser irradiation to induce a 4T1 cell injury (Fig. 3C).

The DNMF/PLGA + laser group was further examined with different laser intensities (0.5, 1.0, and 1.5 W/cm^2^) and NP concentrations (0, 0.05, 0.1, 0.2, and 0.4 mg/mL). Cell survival was negatively correlated with the NP concentration and laser intensity. As the NP concentration and laser intensity increased, the cell survival reduced. When the NP concentration and laser intensity were up to 0.4 mg/mL and 1.5 W/cm^2^, respectively, cell survival was < 20% (Fig. 3D). The in vitro biological safety of DNMF/PLGA NPs was also analyzed in the standard CCK-8 assay by measuring the viability of cells incubated with different concentrations of DNMF/PLGA NPs (Fig. 3E**)**.

To further investigate the therapeutic effect in vitro, the 4T1 cells were treated with seven groups of NPs, as mentioned earlier. The living cells were stained with calcian-AM (green fluorescence), whereas the dead cells were stained with PI (red fluorescence). The DNMF/PLGA + laser group showed the best therapeutic effect after statistical analysis with Image J, the result was consistent with the CCK-8 assay, which further confirmed the inference of synergistic therapy against 4T1 cells (Figs. 3F, Additional file 1: Figure S9A, B).

The FCM was employed to determine the therapeutic effect in vitro. The grouping used was the same as that mentioned above, and the treated 4T1 cells were stained with Annexin V-FITC and PI before FCM analysis. The cellular apoptosis rate induced by DNMF/PLGA NPs + laser was 76.6%, which was significantly higher than that induced by DOX/PLGA (12.58%), N/PLGA (3.59%), and laser only (17%) (Figs. 3G, Additional file 1: Figure S10). The MF/PLGA + laser group demonstrated a good therapeutic effect of 64.4% cellular apoptosis rate. This result is consistent with those of previous experiments implying the excellent photothermal effect of the NPs. Thus, the photothermal effect may play a major role in the synergistic therapy in vitro.

### Mechanistic analysis of DNMF/PLGA NPs in synergistic therapy

**Fig. 4:**
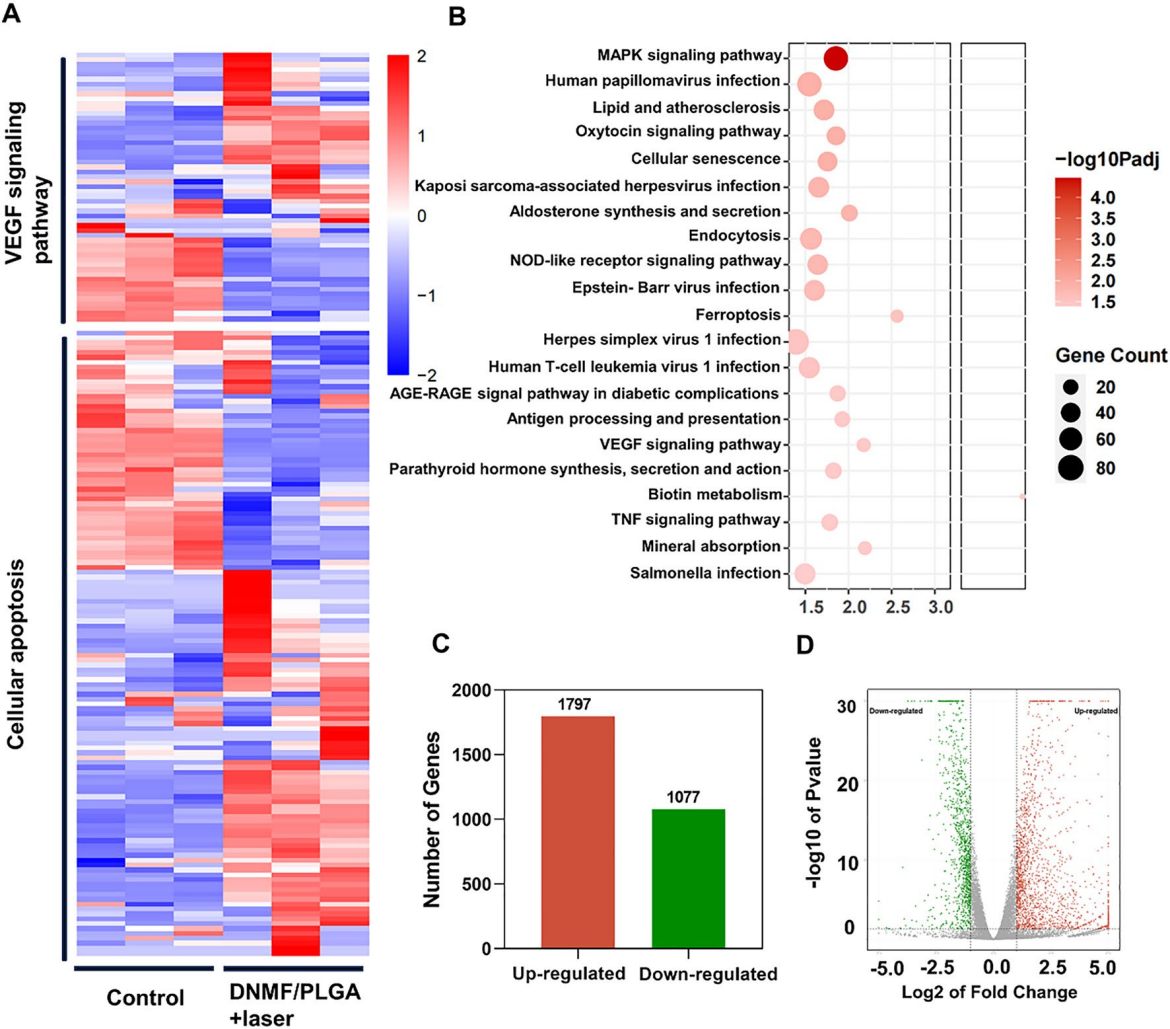
Mechanistic analysis of DNMF/PLGA NPs in combined therapy. **A** Heatmap of the SDEGs associated with the VEGF signaling pathway and apoptosis-related pathways. A warm orange color indicates significantly upregulated genes, while a cold blue color represents remarkably downregulated genes. **B** The volcano plot of SDEGs between the control and DNMF/PLGA NPs + laser groups. Red dots represent upregulated genes, while green dots represent downregulation genes. **C** The number of SDEGs between the control and DNMF/PLGA NPs + laser groups. **D** The KEGG enrichment pathway analysis of SDEGs (fold change ≥ 2.0 (or − 2.0) and P < 0.05)

The potential therapeutic mechanisms of DNMF/PLGA + laser was investigated using the mRNA profiles of the treated 4T1 tumor cells. The 4T1 cells cultured with PBS were used as the control group. Finally, RNA sequencing revealed 2874 significantly differential expressed genes (SDEGs) between the two groups, including 1797 upregulated SDEGs and 1077 downregulated SDEGs (Fig. 4C). A volcano plot based on these SDEGs demonstrated differentially dysregulated genes, suggesting that the expression of these genes was significantly different between the two groups (Fig. 4B). Figure 4A depicts the volcano plot of DEGs. By conducting the Kyoto Encyclopedia of Genes and Genomes analysis, we identified the potential pathways involved in DEGs of TNBC cells, which mainly included apoptosis and VEGF-related pathways (Fig. 4D), as also summarized in Additional file 1: Figure S12. The main mechanisms of 4T1 cell injury were identified as apoptosis and the VEGF signaling pathway [44]. Apoptosis was mainly induced through the upregulation of the photothermal and chemotherapy effects, whereas the upregulation of the VEGF signaling pathway was induced by NO generation, which is consistent with the results of previous experiments [45]. The mechanisms of MMPs activity and the reduction in collagen I concentrations were investigated using mRNA profiles. The result provides a clear understanding of the mechanisms. In tumor, the NPs after irradiation upregulated MMP-1 through the PPAR signaling pathway (Additional file 1: Figure S17A). The upregulated MMP-1 led to an increased activity of MMPs, and the activated MMPs promoted collagen I degradation and the reduction of its concentration, which is a well-known principle. Because collagen is the main component of ECM, the reduction of collagen I concentration resulted in feedback upregulation of ECM through the PI3K-AKT signaling pathway (Additional file 1: Figure S17B), which verified the previous process in the other hand. Previous studies have also confirmed that the activation of these mechanisms originates from the increase concentration of NO [46, 47], which is consistent with our design. Moreover, the main signaling pathways have also been confirmed in previous study [48].

### MRI/PAI/IVIS spectrum CT multimodal imaging in vitro and in vivo

**Fig. 5:**
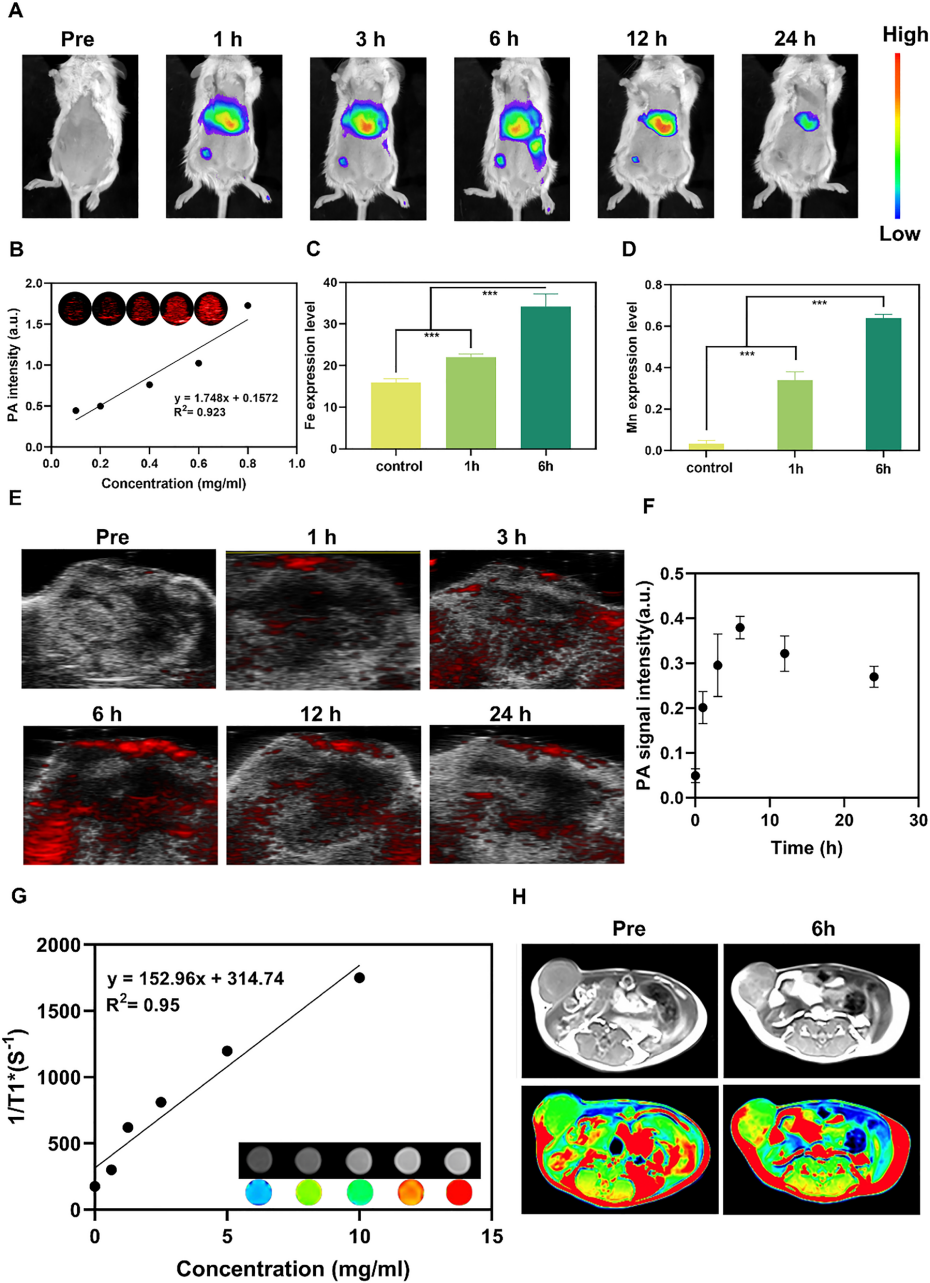
IVIS spectrum CT/MRI/PAI multi-modality imaging in vitro and in vivo. **A** In vivo NIR fluorescence images of tumors in 4T1 tumor-bearing mice after intravenous injection of DNMF/PLGA NPs at different time points. **B** In vitro PAI images and PAI values of DNMF/PLGA NPs at different concentrations. **C** ICP assay of tumors showing the Fe content in the different time points (pre, 1 h, 6 h) after intravenous administration in mice. **D** ICP assay of tumors showing the Mn content at different time points (pre, 1 h, 6 h) after intravenous administration in mice. **E** The average photoacoustic intensity at tumor regions after an intravenous injection of NPs at different time intervals. **F** In vivo PAI images of tumors in 4T1 tumor-bearing mice after intravenous injection of DNMF/PLGA NPs at different time intervals. **G** In vitro MRI T1 contrast images, mappings, and R1* value of DNMF/PLGA at different nanoparticle concentrations (0, 0.625, 1.25, 2.5, 5, and 10 mg/mL). **H** In vivo MRI T1 images of tumors were obtained at 0 and 6 h after an injection of DNMF/PLGA NPs. Data are presented as the means ± SD. Two-way ANOVA with repeated measures with Tukey’s post-hoc test. *p < 0.05

The accumulation of NPs in 4T1 cells was also detected in vitro. The IVIS spectrum CT multimodal imaging system was employed to examine the accumulation of DIR-labeled NPs in the 4T1 cells. The DIR signal values enhanced linearly with an increase in the concentration from 0.5 to 2.5 mg/mL (Additional file 1: Figure S11A). The fluorescent images were also detected in vivo. Fluorescence appeared at 1 h, peaked at 6 h, and then declined after 6 h (Figs. 5A, Additional file 1: Figure S11D). The quantitative analysis also confirmed the results (Additional file 1: Figure S11C). The observation time continued to 24 h. The anatomical liver, spleen, heart, kidney, lung, and tumor were also detected, and the fluorescence of the liver and spleen still can be inspected (Additional file 1: Figure S11B). After the intravenous injection of DIR-labeled NPs, the tumors were removed to detect the contents of Fe and Mn elements at different time points. As a result, the Fe and Mn elements in tumors at 6 h were significantly higher than those at pre and 1 h (Fig. 5C, D), which indicated the gradual accumulation of NPs in tumors for possible further PTT and imaging.

Because of the prominent photothermal conversion performance of DNMF/PLGA NPs, in vitro and in vivo PA images were acquired using a Vevo LAZR PA imaging system. Being a desirable PAI agent, DNMF/PLGA NPs exhibited excellent PAI contrast enhancement in vitro. The optimal wavelengths of 680–690 nm were observed for DNMF/PLGA NPs, which is similar to those in other studies of MnFe_2_O_4_. PAI signal values increased linearly with an increase in the concentrations from 0.1 to 0.8 mg/mL in vitro (Fig. 5B). Following intravenous injection, the NPs reached the tumor area for PAI at 1 h, peaked at 6 h, and then declined after 6 h, which is similar to that in fluorography (Fig. 5E). The quantitative analysis also confirmed the results (Fig. 5F). However, different from fluorography, the PAI signal was detected even at 24 h, which may be because of the difference in the sensitivity of different imaging methods. In vivo PAI results suggested that DNMF/PLGA NPs can accumulate at the tumor site through their passive targeting behavior as well as have an appropriate residence time of at least 1–12 h, which would be beneficial for photothermal therapy, DOX release, NO generation, and real-time observation.

As the most accurate non-invasive single-imaging technology, MRI is crucial for breast cancer diagnosis [49]. Herein, MRI T1 imaging of DNMF/PLGA NPs was systematically investigated. As shown in Fig. 5G, the brightness of MRI contrast images of DNMF/PLGA NPs increased linearly with an increase in the NP concentration in vitro. Following an intravenous injection of DNMF/PLGA NPs, an outstanding T1 signal enhancement was observed at 6 h in vivo (Fig. 5H). Based on the mapping technology of MRI, the enhanced tumor imaging was more clearly represented [50]. Thus, the NPs can be used as a fast and accurate MRI-contrast agent.

### In vitro biocompatibility and in vivo biosafety assay

In vitro biocompatibility of DNMF/PLGA NPs was investigated by measuring the viability of cells co-incubated with DNMF/PLGA NPs in the standard CCK-8 assay (Fig. 3E). Slight significant cytotoxicity was noted toward 4T1 cells even at 0.8 mg/mL for the 48-h co-incubation duration.

**Fig. 6:**
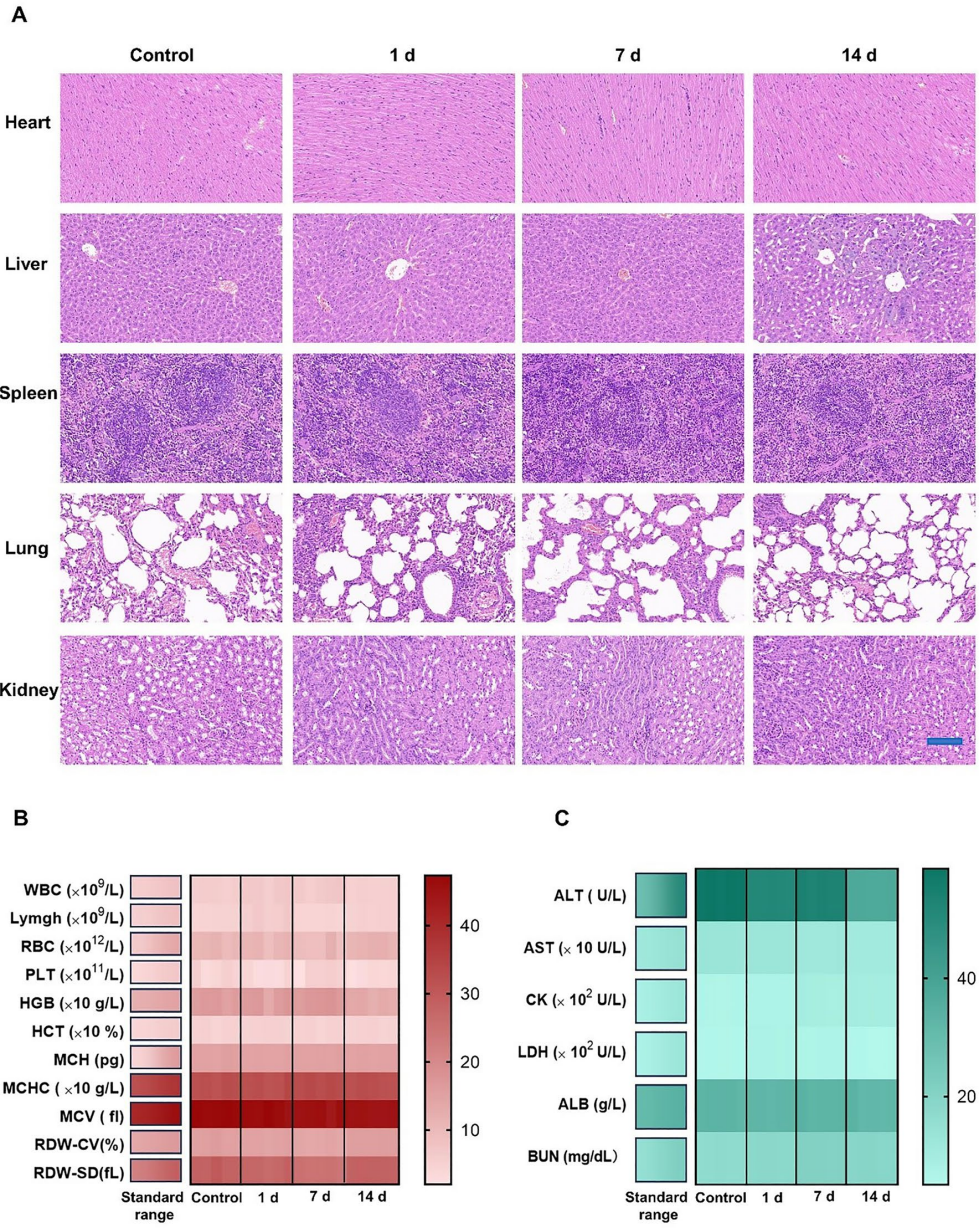
In vitro biocompatibility and in vivo biosafety assay. **A** H&E staining of the major organ tissues. **B** Routine blood tests. **C** Blood biochemistry analysis. All of the abovementioned examinations were performed after an intravenous injection with PBS and DNMF/PLGA NPs (1, 7, and 14 days). Data are presented as the mean ± SD

In vivo biosafety was systematically investigated for the short and long terms. The healthy mice were intravenously injected with DNMF/PLGA NPs, while the other mice that received no treatment were included as the control group. Blood was collected from the mice for biochemical examination (including liver and kidney functions) and routine blood analysis at pretreatment and then at 1, 7, and 14 days. Vital organs (including the liver, spleen, heart, kidney, and lung) were dissected for pathological examination at the same time point. When compared with the control group, no significant variation in biochemical or routine blood analyses was noted at 1, 7, and 14 days (Fig. 6B, C). Furthermore, the dissected major organs were sectioned for H&E staining, which revealed no significant histopathological changes at least in 14 days **(**Fig. 6A). For detail, the normal cardiomyocytes, alveolar epithelial cells and alveolar structures, liver lobular structures, glomerulus structures, and splenic cord were clearly identified without obvious pathological changes at 1,7, and 14 days.

These results demonstrated that DNMF/PLGA NPs have good biocompatibility in vitro and biosafety in vivo, which is the essential prerequisite for clinical translation.

### In vivo antitumor effect of DNMF/PLGA NPs

**Fig. 7:**
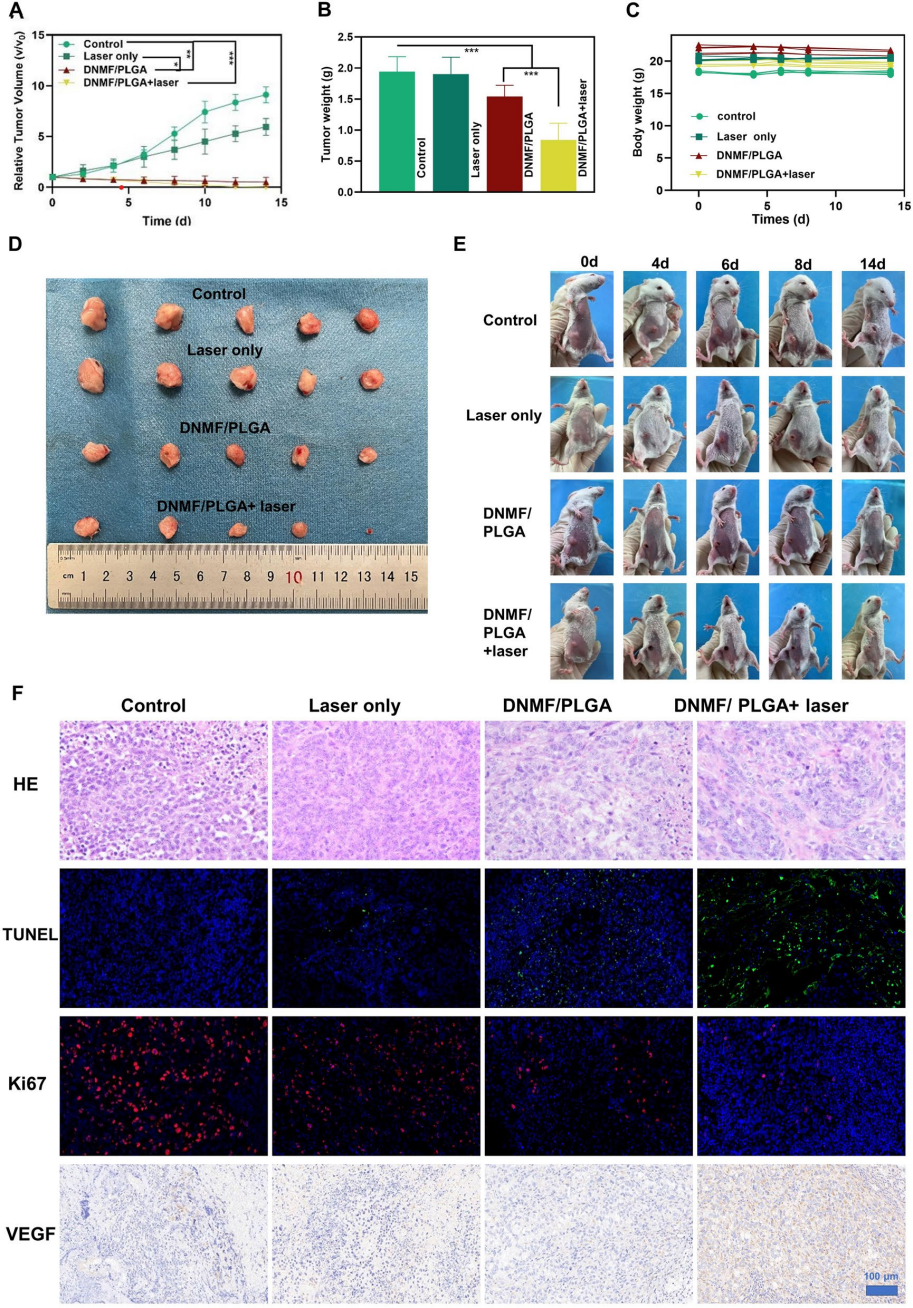
In vivo anti-tumor effect of DNMF/PLGA NPs. **A** The relative tumor growth curves from 0 to 14 days. **B** The ex vivo tumor weights after different treatments at 14 days. **C** Body weight of 4T1 tumor-bearing mice in different groups. **D** Images of ex vivo 4T1 tumors after receiving different treatments at 14 days. **E** Images of in vivo 4T1 tumors after receiving different treatments at different time points. **F** H&E, TUNEL, VEGF, and Ki-67 antibody staining images of tumor tissues in different treatment groups. n = 5, mean ± SD. Two-way ANOVA with repeated measures with Tukey’s post-hoc test. *p < 0.05

The in vivo effects of synergistic photothermal and chemotherapy on tumor vessel normalization were evaluated in a TNBC mouse model. The mice were randomly assigned to four treatment groups, namely control, laser only, DNMF/PLGA NPs, and DNMF/PLGA NPs + laser. When the tumors grew to approximately 1 cm^3^, the mice received different treatments as per the group they belonged to. The mice in the laser groups were irradiated with an 808-nm laser for 10 min. The DNMF/PLGA NPs + laser group was irradiated at 6 h after intravenous injection based on the previous experiments. The body weights of the mice were recorded during the 14 days, while the tumor weights were recorded at 14 days. The body weights exhibited no change, but the tumor weights significantly differed between the DNMF/PLGA NPs + laser and other groups. The DNMF/PLGA NPs + laser group had the minimum tumor weight, indicating it had the best therapeutic effect. The DNMF/PLGA NP group exhibited a certain effect because of the slight natural lysis of the NPs and drug release, while the laser-only group had little effect without any photothermal conversion materials (Fig. 7B, C). After different treatments, all groups were monitored every 2 days during the entire observation period. As shown in Fig. 7A, the volumes of tumors dynamically changed over time, and the changes were consistent with the expectations. The same trend was also observed through tumor variations in vitro and in vivo (Fig. 7D, E). Furthermore, the therapeutic effects after various treatments were determined with H&E, TUNEL, Ki-67, and VEGF staining (Fig. 7F). The prominent apoptosis/necrosis of tumor tissues was observed in the DNMF/PLGA + laser group, while a certain inhibition effect was observed in the DNMF/PLGA group, which is consistent with the previous experiment results. The VEGF expression was upregulated for tumor vessel normalization, which could increase the NP delivery [51]. However, considering the possible risk of tumor metastasis noted in some past studies [52], we did not encourage NO-induced vessel normalization without the delivery of effective therapeutic NPs. In summary, synergetic therapy exhibited a surprising effect of inhibiting TNBC tumors through different mechanisms.

### NO effect of vascular normalization in tumor tissues

**Fig. 8:**
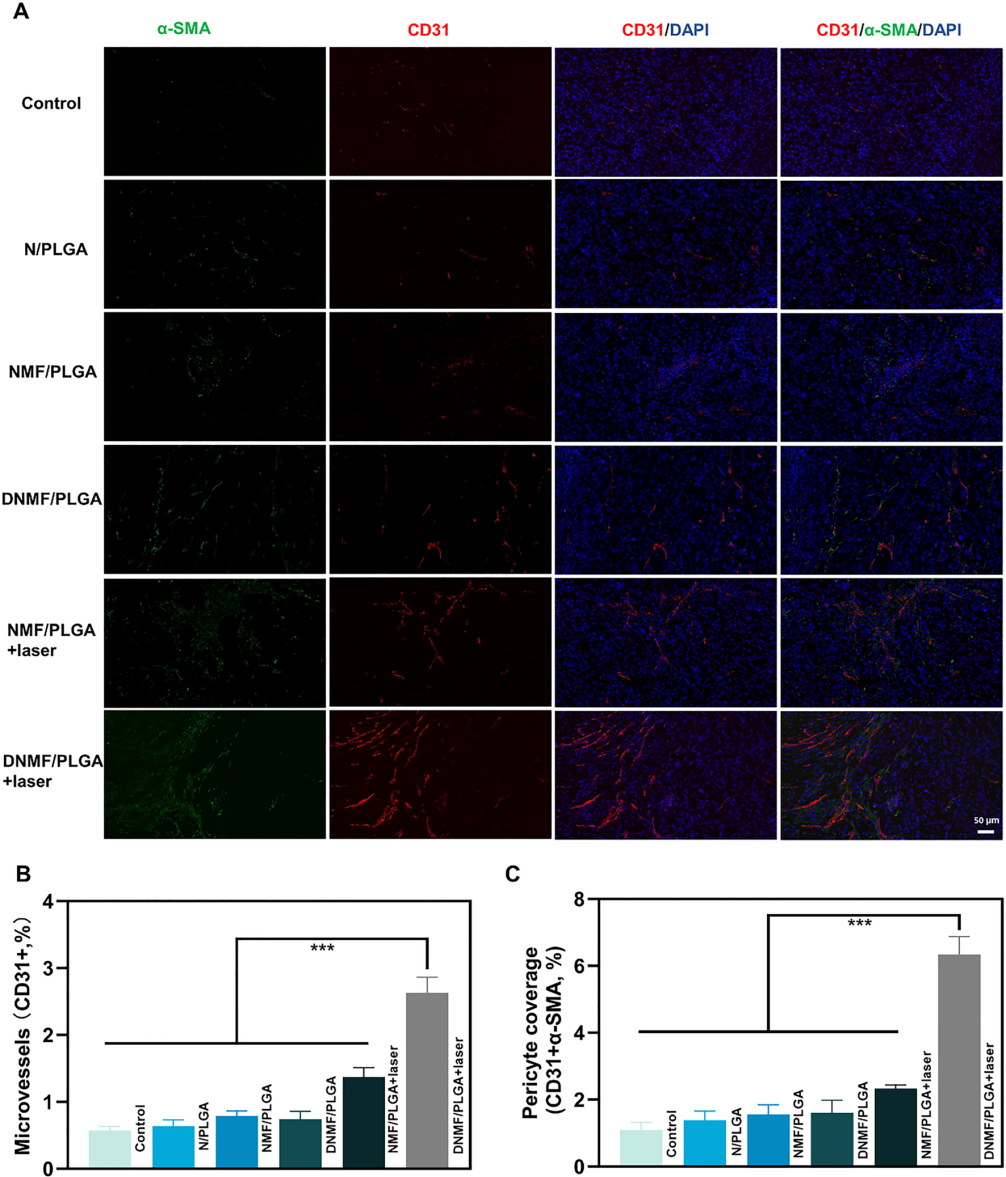
The effect of tumor vessel normalization. **A** The immunofluorescence images of CD31 + and α-SMA, and the immunofluorescence images of co-stained CD31 + and α-SMA in the tumor after different treatments. CD31 + endothelial cells are stained red, and α-SMA pericytes are stained green. The experiments were repeated at least thrice. **B**, **C** Images from immunofluorescence were quantified with Image J

The mean vascular density (MVD) and pericyte coverage of tumor vascular are commonly used for evaluating tumor vascular normalization [53]. We employed CD31 staining and co-staining of CD31 + and alpha-smooth muscle actin (α-SMA) to evaluate the vascular structure and verify the effect of the NO generated from NPs in the tumor vascular environment. The expression of CD31 and α-SMA significantly increased in the DNMF/PLGA + laser group (Fig. 8A), whereas a low expression level was noted in the control group. Furthermore, a denser network of capillaries was observed in the DNMF/PLGA + laser group, whereas a sparse and scattered distribution of capillaries was observed in the control group, which is a significant feature of vessel normalization. Further quantitative analysis revealed that microvessels (CD31 +) and pericyte coverage (CD31 + α-SMA) significantly increased in the DNMF/PLGA + laser group, which also confirmed the results (Fig. 8B, C). In summary, by combining the present results with previous experimental results, we concluded that NO-induced vessel normalization and improvement in the tumor microenvironment mainly included an increase in the functional vessels and collagen degradation, which are beneficial for NP delivery, drug penetration, and related therapies.

## Conclusions

We reported the use of novel laser-triggered multimodality multifunctional NPs for TNBC treatment. The NPs could enhance MRI/PAI, which was beneficial for the early diagnosis and treatment monitoring of TNBC. Following laser irradiation, the DOX and MnFe_2_O_4_ released from the NPs served as chemo-hyperthermia synergistic therapeutic agents against TNBC, while the released L-Arg acted as a NO donor for NO-induced tumor normalization and extravascular collagen degradation. We proposed a tumor vascular microenvironment remodeling strategy as an auxiliary mechanism for TNBC treatment. The chemo-hyperthermia synergetic inhibition mechanism and the auxiliary mechanism were perfectly matched to exert excellent effects against TNBC. Although previous studies reported the good tumor therapeutic effects, we still reiterate the limitation that we did not encourage NO-induced vessel normalization without the delivery of effective therapeutic NPs. However, the potential risks and benefits are indeed issues that deserves further comprehensive and in-depth exploration in future. In brief, the novel NPs offer an interesting composite strategy, thereby laying the foundation for future research to address the clinical needs of TNBC.

### Supplementary Information

Additional file 1: **Figure S1. **Zeta potential of the DNMF/PLGA NPs within 7 days. Data are presented as the means ±SD. ANOVA with Dunnett's post-hoc test. **Figure S2.** Size distribution of the DNMF/PLGA NPs within 7 days. Data are presented as the means ±SD. ANOVA with Dunnett's post-hoc test. **Figure S3.** Size distribution of the N/PLGA, DOX/PLGA, MF/PLGA, and DNMF/PLGA NPs. **Figure S4.** A) Encapsulating efficiency and B) loading capacity of MnFe_2_O_4_ in the DNMF/PLGA NPs with different initial MnFe_2_O_4_ loadings (20, 40, 80, and 160 µL). Data are presented as the means ±SD. A–D) ANOVA with Dunnett's post-hoc test. *** p < 0.001. **Figure S5.** After 4T1 cells were treated with five different groups, the H_2_O_2_ was quantitatively tested using a H_2_O_2_ detection kit. **Figure S6.** A) CLSM images and B) FCM analysis of FITC-labeled ROS in six different groups after treatments for 4 h. C) Quantitative analysis of the ROS by FCM analysis intensity. Data are presented as the means ±SD. ANOVA with Tukey’s post-hoc test. *p < 0.05, **p < 0.01, and ***p < 0.001. **Figure S7.** A) Quantitative analysis of the NO level in 4T1 cells detected by using CLSM. B) Quantitative analysis of the NO level in 4T1 cells detected by using FCM. C) Quantitative analysis of immunofluorescent staining of NO release in 4T1 tumor. A, B, C) ANOVA with Tukey’s post-hoc test. *p < 0.05, **p < 0.01, and ***p < 0.001. **Figure S8.** After 4T1 cells were incubated for 0, 0.5, 1,2, 4 h, the intracellular uptake of nanoparticles was observed using FCM. Corresponding quantitative analysis evaluated by FCM. ANOVA with Tukey’s post-hoc test. *p < 0.05, **p < 0.01, and ***p < 0.001. **Figure S9.** A) FCM apoptosis assay of 4T1 cells stained by Annexin-FITC and PI after different treatments. The power density was 1.5 W cm^−2^ and the irradiation time was 5 min. Apoptosis rate evaluated by FCM. B) Quantitative analysis of the live-death level in 4T1 cells detected by using CLSM.ANOVA with Tukey’s post-hoc test. *p < 0.05, **p < 0.01, and ***p < 0.001. **Figure S10.**A) Quantitative analysis of the 3-NT intensity. B) Quantitative analysis of the collagen fluorescence intensity. Data are presented as the means ±SD. A, B) ANOVA with Tukey’s post-hoc test. *p < 0.05, **p < 0.01, and ***p < 0.001. **Figure S11. **A) In vivo fluorescence images of DNMF/PLGA NPs at different concentrations. B) *In vivo* fluorescence images of *ex vivo* major organs after 24 h of DNMF/PLGA NPs intravenous injection. C) The variations of fluorescence signal intensities within tumor regions at the corresponding time points. D) Corresponding quantitative analysis of fluorescence intensity of tumor in vivo. Two-way ANOVA with repeated measures using Tukey’s post-hoc test. ANOVA with Tukey’s post-hoc test. *p < 0.05, **p < 0.01, and ***p < 0.001. **Figure S12. **Mechanistic analysis of DNMF/PLGA NPs in combined therapy. The volcano plot of apoptosis-related pathways and VEGF-related pathways. **Figure S13.** A) Zeta potential of the DNMF/PLGA NPs within 7 days. B) Size distribution of the DNMF/PLGA NPs within 7 days. Data are presented as the means ±SD. ANOVA with Dunnett's post-hoc test. **Figure S14. **TEM image of the DNMF/PLGA NPs. **Figure S15. **The amount of DOX loading and encapsulation, the amount of L-Arg loading and encapsulation (n = 3). **Figure S16. **A) Following 808-nm laser irradiation for 5 min, Mn^2+^ and Fe^2+^release in physiological state was observed at time point of 0h, 1h, 6h, 12h, 24h, 48h. B) Without laser irradiation. **Figure S17.** Mechanistic analysis of MMPs activity and collagen I reduction. A) MMP-1 target gene data on KEGG graph rendered by pathview. B) Collagen I related gene data on KEGG graph rendered by pathview. A warm red color indicates significantly upregulated genes, while a cold green color represents remarkably downregulated genes. **Table S1.** The comparison of NO-reactor nanoparticles.

## Data Availability

The data that support the findings of this study are available on request from the corresponding author. The data are not publicly available due to privacy or ethical restrictions.
