# Supplementary material for: Nitric oxide nano-reactor DNMF/PLGA enables tumor vascular microenvironment and chemo-hyperthermia synergetic therapy
Source: J Nanobiotechnology. 2024 Mar 13;22:110. doi: 10.1186/s12951-024-02366-y (PMC10938667; doi:10.1186/s12951-024-02366-y)
Supplement: Supplementary file 1 — Additional file 1: Figure S1. Zeta potential of the DNMF/PLGA NPs within 7 days. Data are presented as the means ±SD. ANOVA with Dunnett's post-hoc test. Figure S2. Size distribution of the DNMF/PLGA NPs within 7 days. Data are presented as the means ±SD. ANOVA with Dunnett's post-hoc test. Figure S3. Size distribution of the N/PLGA, DOX/PLGA, MF/PLGA, and DNMF/PLGA NPs. Figure S4. A) Encapsulating efficiency and B) loading capacity of MnFe2O4 in the DNMF/PLGA NPs with different initial MnFe2O4 loadings (20, 40, 80, and 160 µL). Data are presented as the means ±SD. A–D) ANOVA with Dunnett's post-hoc test. *** p < 0.001. Figure S5. After 4T1 cells were treated with five different groups, the H2O2 was quantitatively tested using a H2O2 detection kit. Figure S6. A) CLSM images and B) FCM analysis of FITC-labeled ROS in six different groups after treatments for 4 h. C) Quantitative analysis of the ROS by FCM analysis intensity. Data are presented as the means ±SD. ANOVA with Tukey’s post-hoc test. *p < 0.05, **p < 0.01, and ***p < 0.001. Figure S7. A) Quantitative analysis of the NO level in 4T1 cells detected by using CLSM. B) Quantitative analysis of the NO level in 4T1 cells detected by using FCM. C) Quantitative analysis of immunofluorescent staining of NO release in 4T1 tumor. A, B, C) ANOVA with Tukey’s post-hoc test. *p < 0.05, **p < 0.01, and ***p < 0.001. Figure S8. After 4T1 cells were incubated for 0, 0.5, 1,2, 4 h, the intracellular uptake of nanoparticles was observed using FCM. Corresponding quantitative analysis evaluated by FCM. ANOVA with Tukey’s post-hoc test. *p < 0.05, **p < 0.01, and ***p < 0.001. Figure S9. A) FCM apoptosis assay of 4T1 cells stained by Annexin-FITC and PI after different treatments. The power density was 1.5 W cm−2 and the irradiation time was 5 min. Apoptosis rate evaluated by FCM. B) Quantitative analysis of the live-death level in 4T1 cells detected by using CLSM.ANOVA with Tukey’s post-hoc test. *p < 0.05, **p < 0.01, and ***p [file 12951_2024_2366_MOESM1_ESM.docx]

Additional Information

Nitric oxide nano-reactor DNMF/PLGA enables tumor vascular microenvironment and chemo-hyperthermia synergetic therapy

*Ruoyao Wang, Long Cheng, Lingyun He, Chier Du, Haiyang Wang, Bohao Peng, Xiaoqing Yu, Weiwei Liu, Wenpei Luo, Haitao Ran, Lu Yang^*^*

R. Wang, B. Peng, X. Yu, L. Yang

Department of Breast and Thyroid Surgery

The Second Affiliated Hospital of Chongqing Medical University

Chongqing 400010, P. R. China

1. mail: [302118@cqmu.edu.cn](mailto:302118@cqmu.edu.cn)

L. Cheng, L. He, C. Du, H. Wang, W. Liu, W. Luo, H. Ran

Department of Ultrasound

The Second Affiliated Hospital of Chongqing Medical University

Chongqing 400010, P. R. China


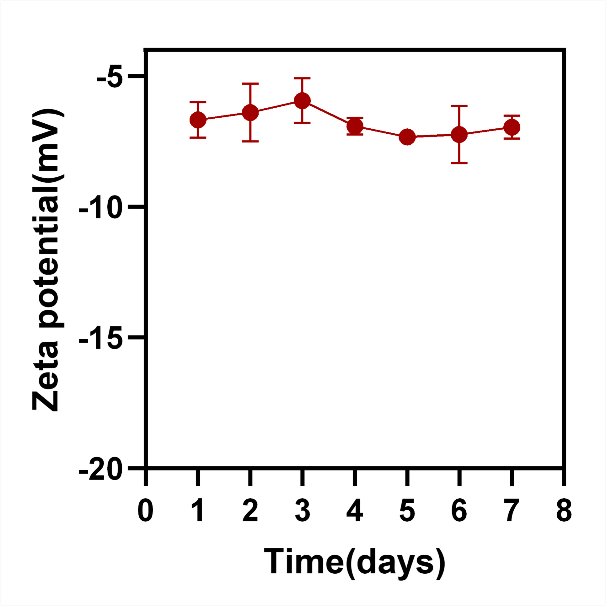


**Figure S1.** Zeta potential of the DNMF/PLGA NPs within 7 days. Data are presented as the means ±SD. ANOVA with Dunnett's post-hoc test.


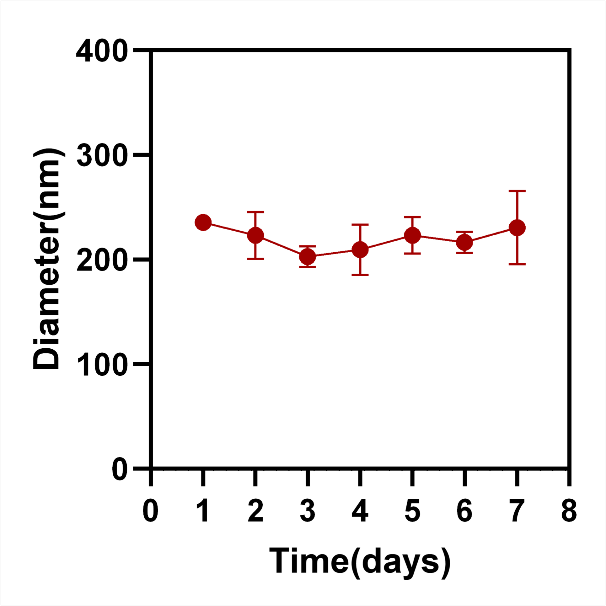


**Figure S2.** Size distribution of the DNMF/PLGA NPs within 7 days. Data are presented as the means ±SD. ANOVA with Dunnett's post-hoc test.


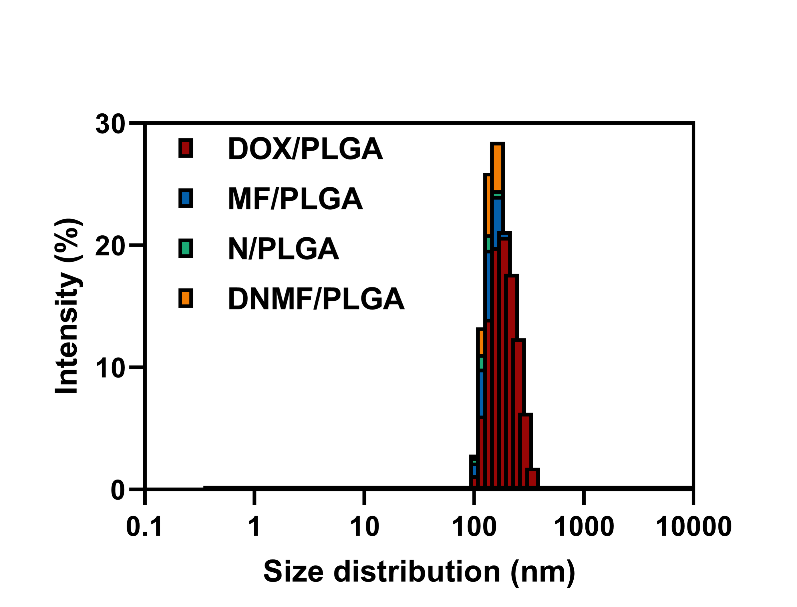


**Figure S3.** Size distribution of the N/PLGA, DOX/PLGA, MF/PLGA, and DNMF/PLGA NPs.


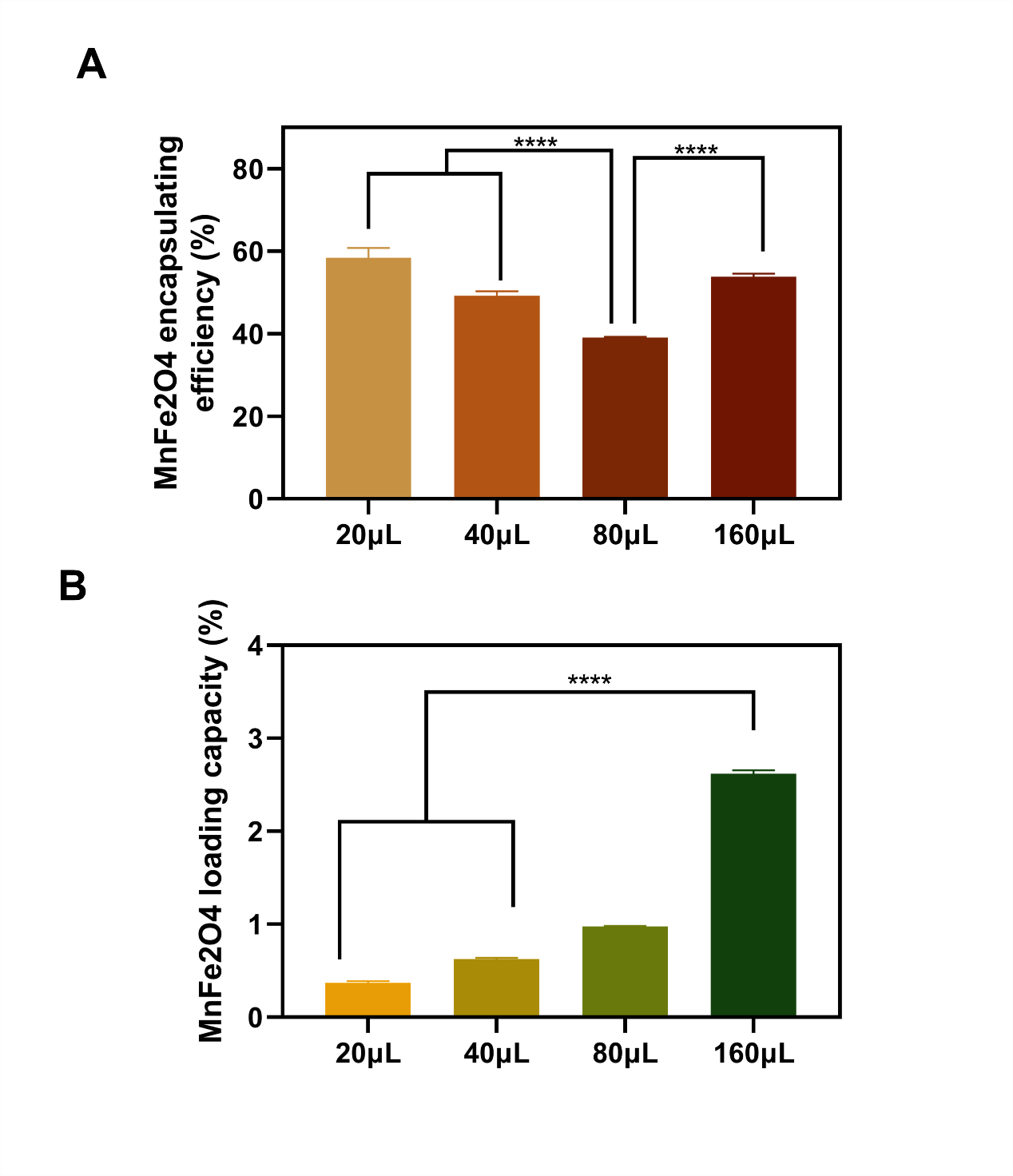


**Figure S4.** A) Encapsulating efficiency and B) loading capacity of MnFe_2_O_4_ in the DNMF/PLGA NPs with different initial MnFe_2_O_4_ loadings (20, 40, 80, and 160 µL). Data are presented as the means ±SD. A–D) ANOVA with Dunnett's post-hoc test. *** p < 0.001


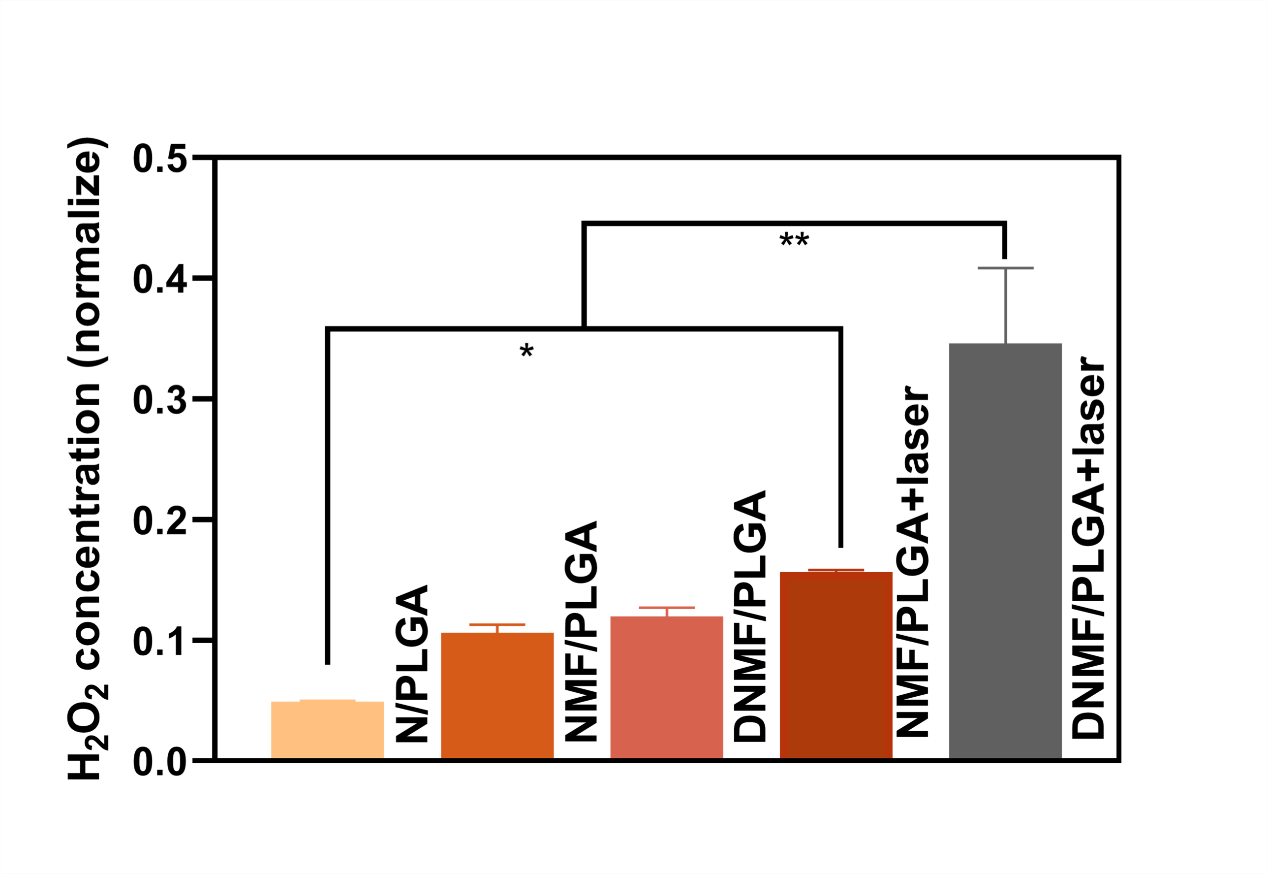


**Figure S5.** After 4T1 cells were treated with five different groups, the H_2_O_2_ was quantitatively tested using a H_2_O_2_ detection kit.


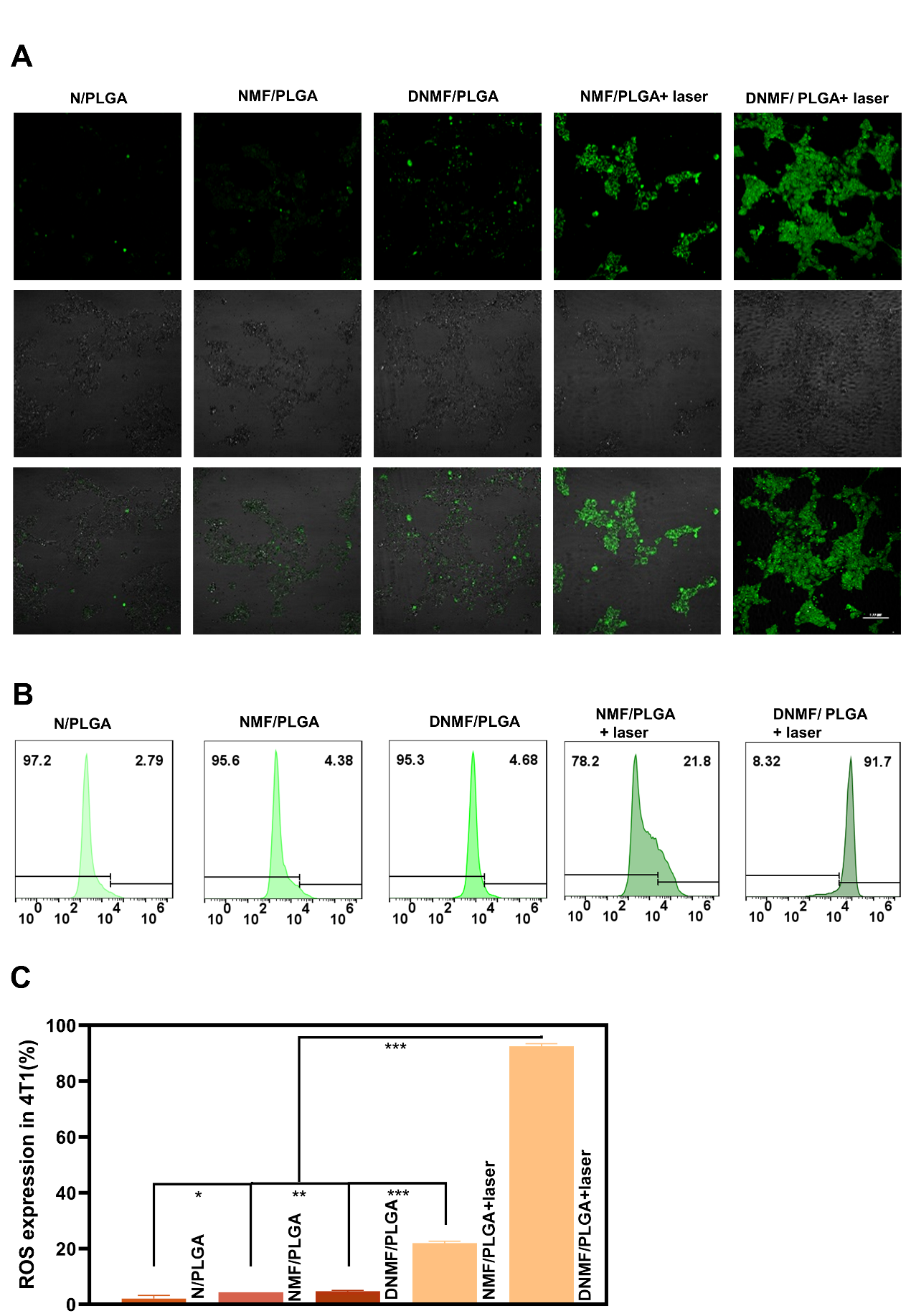


**Figure S6.** A) CLSM images and B) FCM analysis of FITC-labeled ROS in six different groups after treatments for 4 h. C) Quantitative analysis of the ROS by FCM analysis intensity. Data are presented as the means ±SD. ANOVA with Tukey’s post-hoc test. *p < 0.05, **p < 0.01, and ***p < 0.001.


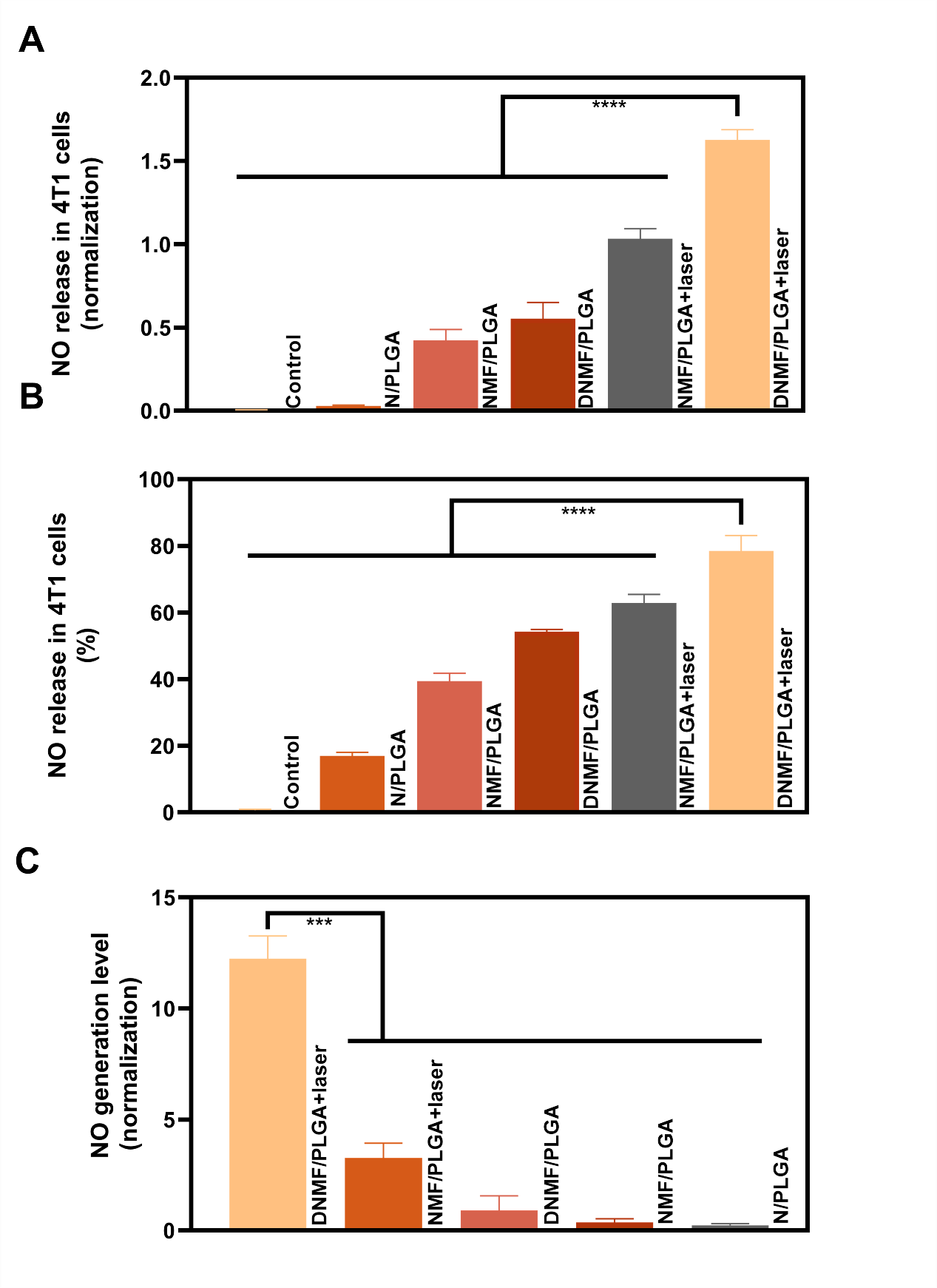


**Figure S7.** A) Quantitative analysis of the NO level in 4T1 cells detected by using CLSM. B) Quantitative analysis of the NO level in 4T1 cells detected by using FCM. C) Quantitative analysis of immunofluorescent staining of NO release in 4T1 tumor. A, B, C) ANOVA with Tukey’s post-hoc test. *p < 0.05, **p < 0.01, and ***p < 0.001.


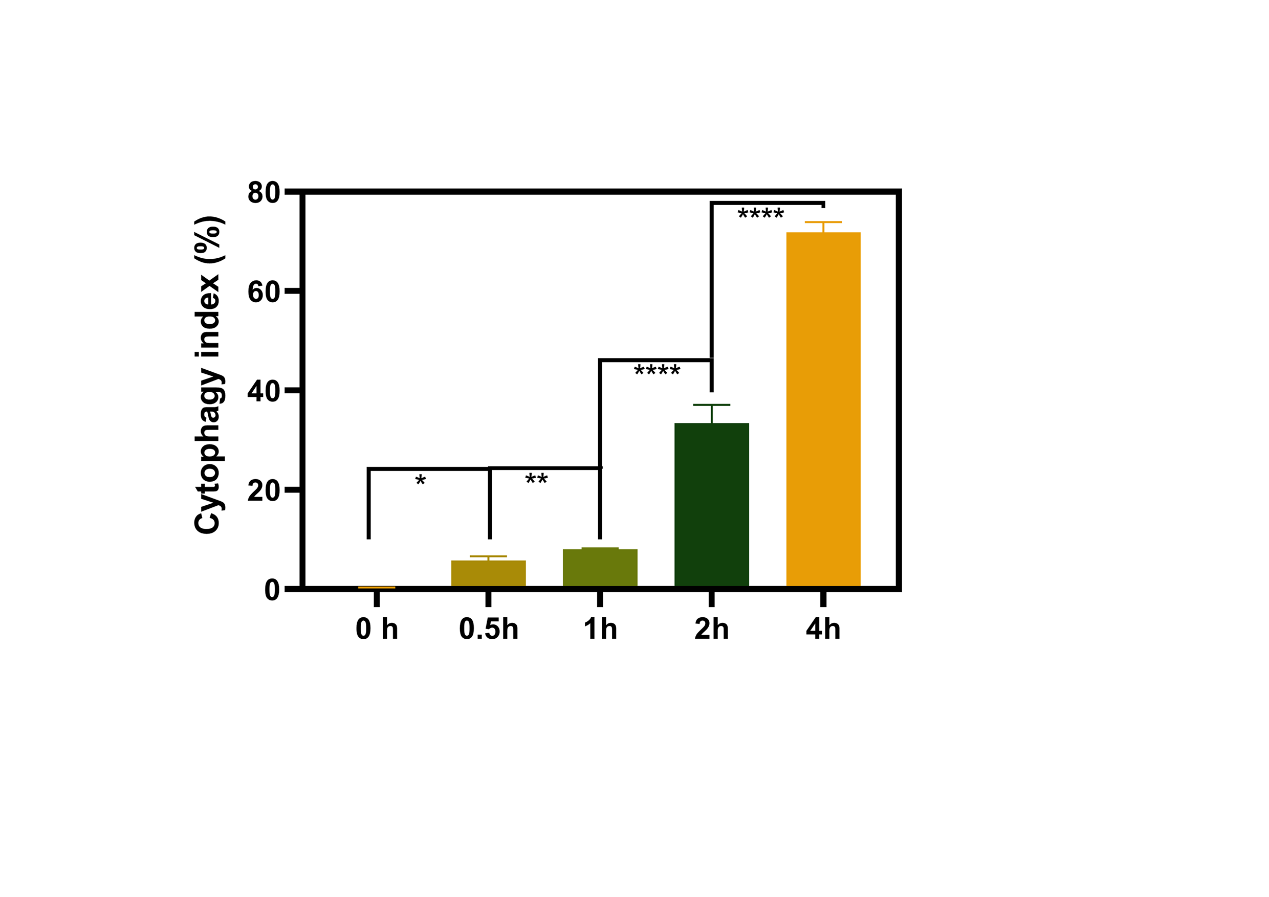


**Figure S8.** After 4T1 cells were incubated for 0, 0.5, 1,2, 4 h, the intracellular uptake of nanoparticles was observed using FCM. Corresponding quantitative analysis evaluated by FCM. ANOVA with Tukey’s post-hoc test. *p < 0.05, **p < 0.01, and ***p < 0.001.


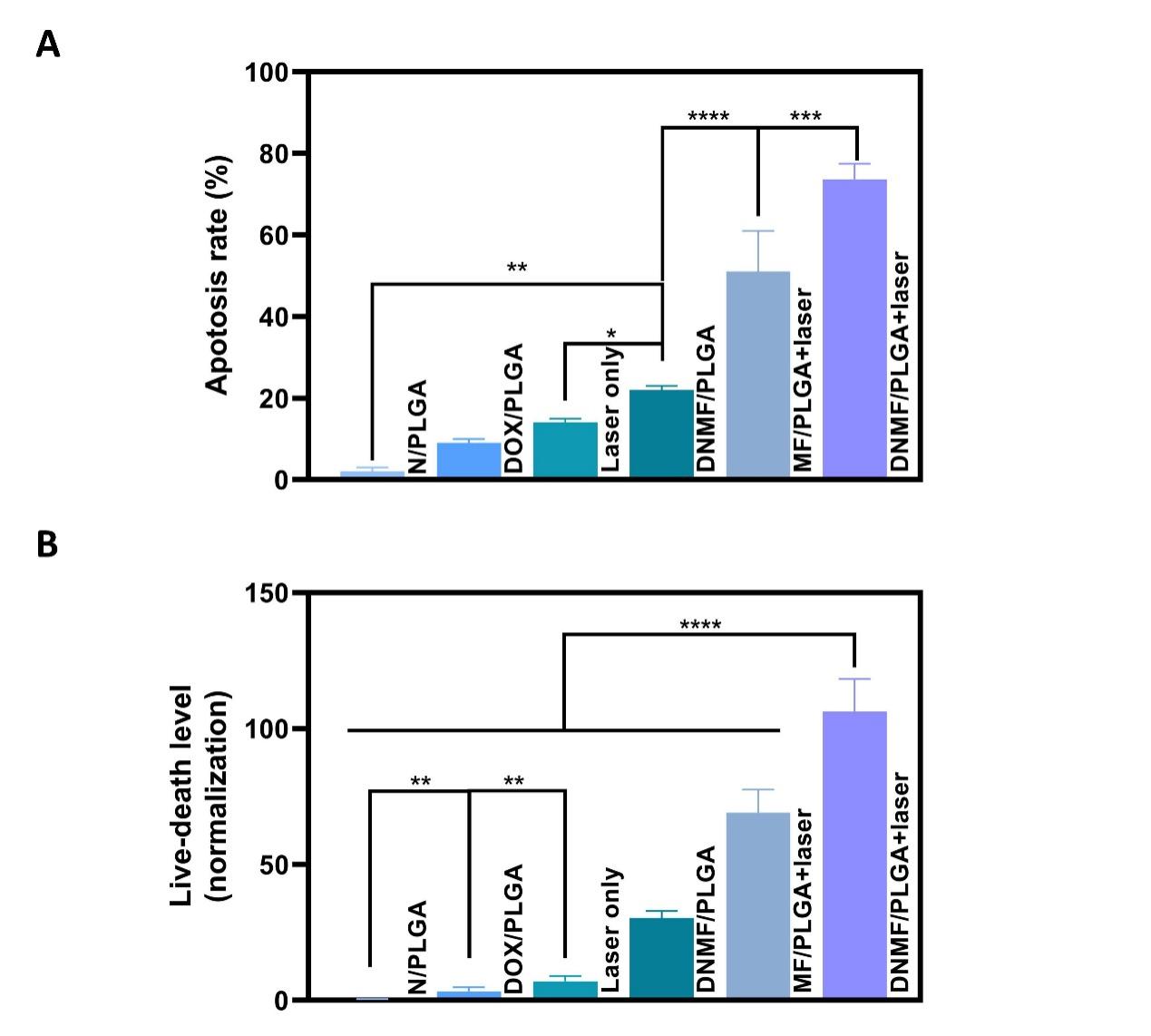


**Figure S9.** A) FCM apoptosis assay of 4T1 cells stained by Annexin-FITC and PI after different treatments. The power density was 1.5 W cm^−2^ and the irradiation time was 5 min. Apoptosis rate evaluated by FCM. B) Quantitative analysis of the live-death level in 4T1 cells detected by using CLSM.ANOVA with Tukey’s post-hoc test. *p < 0.05, **p < 0.01, and ***p < 0.001.


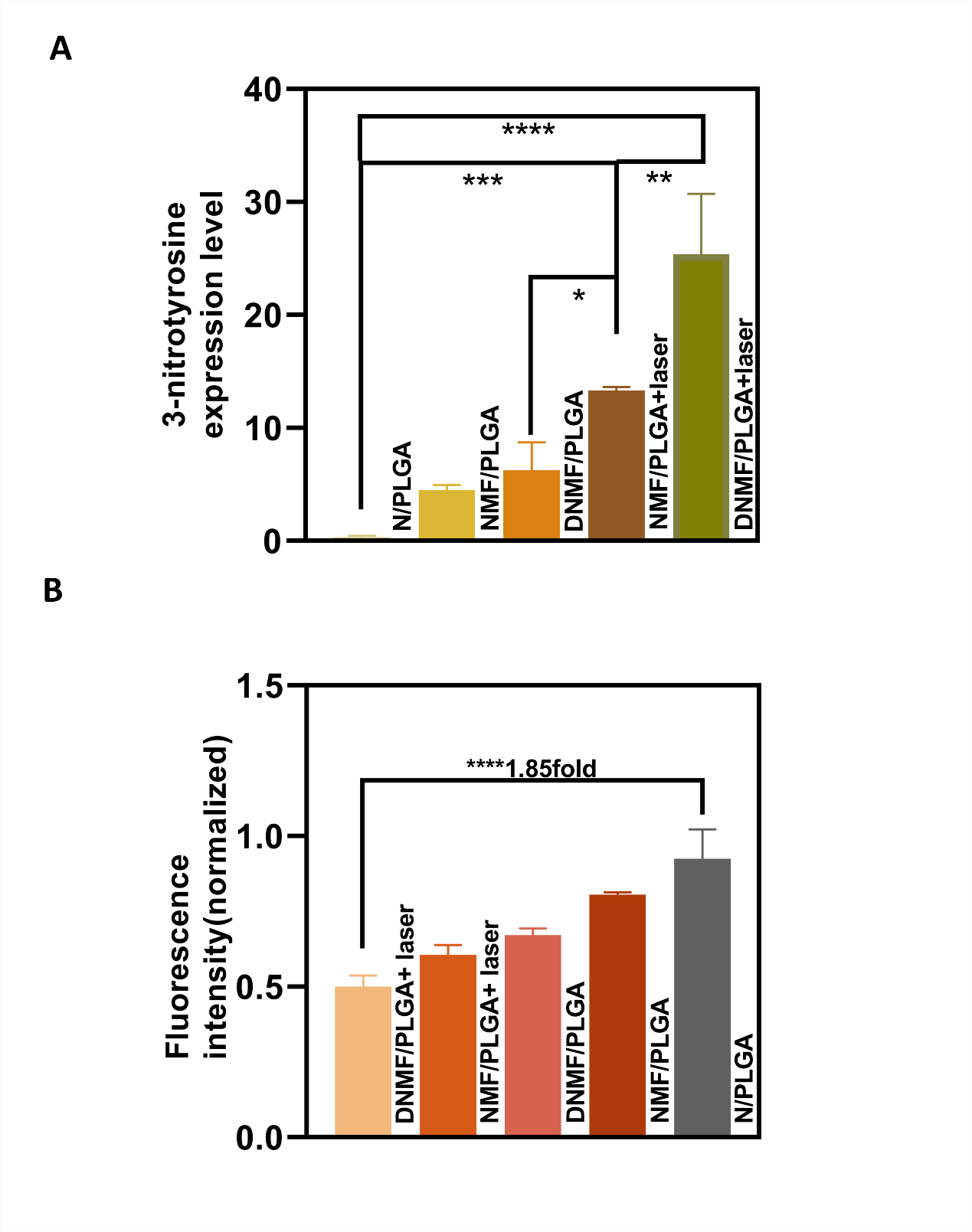


**Figure S10.**A) Quantitative analysis of the 3-NT intensity. B) Quantitative analysis of the collagen fluorescence intensity. Data are presented as the means ±SD. A, B) ANOVA with Tukey’s post-hoc test. *p < 0.05, **p < 0.01, and ***p < 0.001.


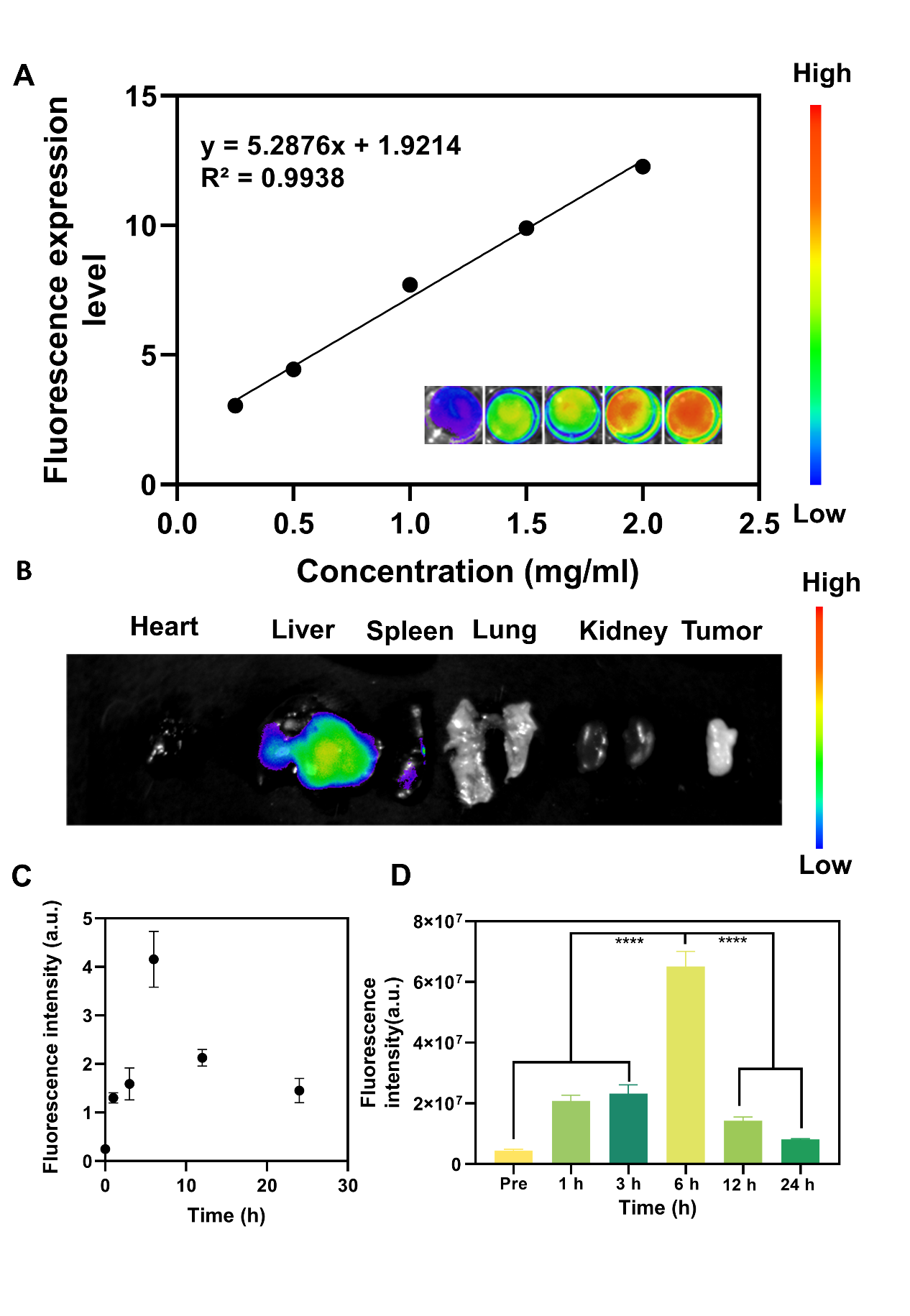


**Figure S11.** A) In vivo fluorescence images of DNMF/PLGA NPs at different concentrations. B) *In vivo* fluorescence images of *ex vivo* major organs after 24 h of DNMF/PLGA NPs intravenous injection. C) The variations of fluorescence signal intensities within tumor regions at the corresponding time points. D) Corresponding quantitative analysis of fluorescence intensity of tumor in vivo. Two-way ANOVA with repeated measures using Tukey’s post-hoc test. ANOVA with Tukey’s post-hoc test. *p < 0.05, **p < 0.01, and ***p < 0.001.


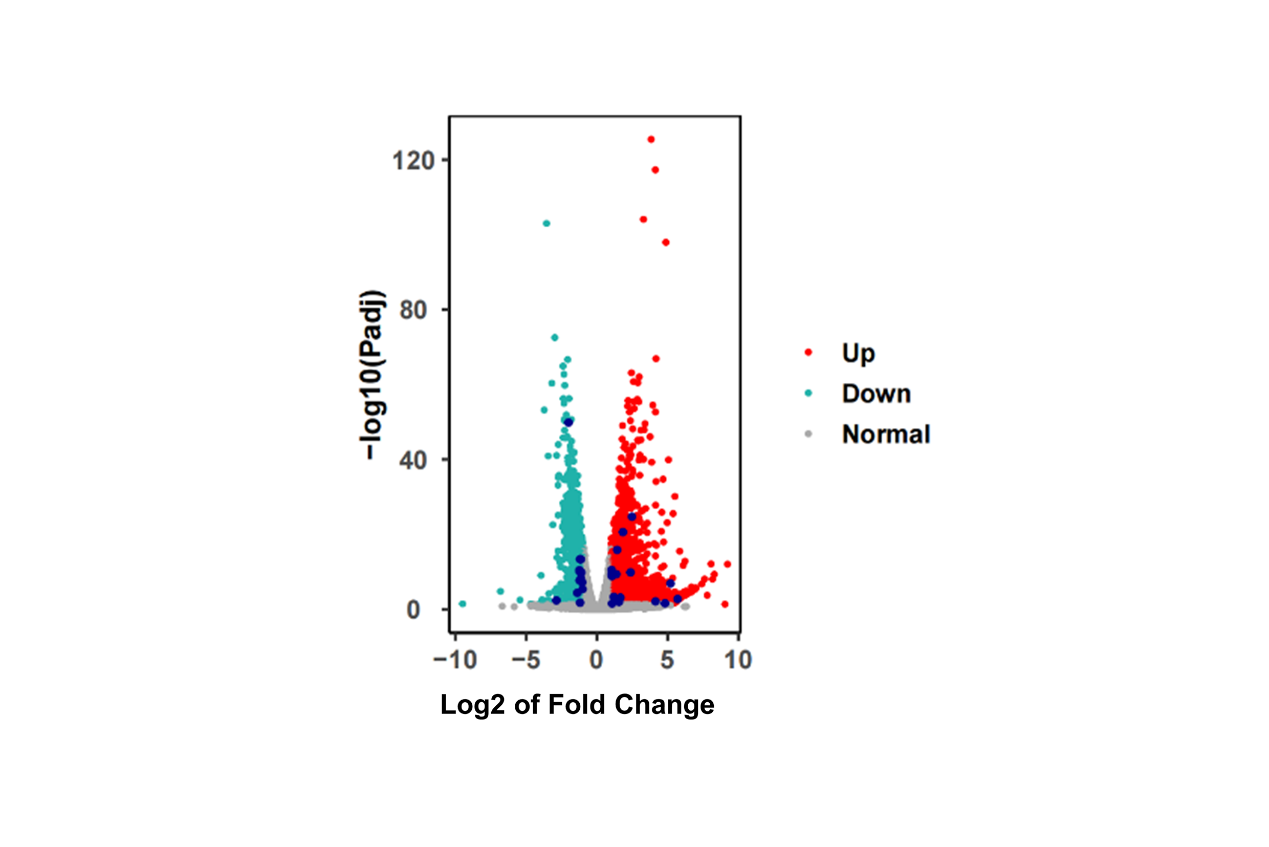


**Figure S12.** Mechanistic analysis of DNMF/PLGA NPs in combined therapy. The volcano plot of apoptosis-related pathways and VEGF-related pathways.


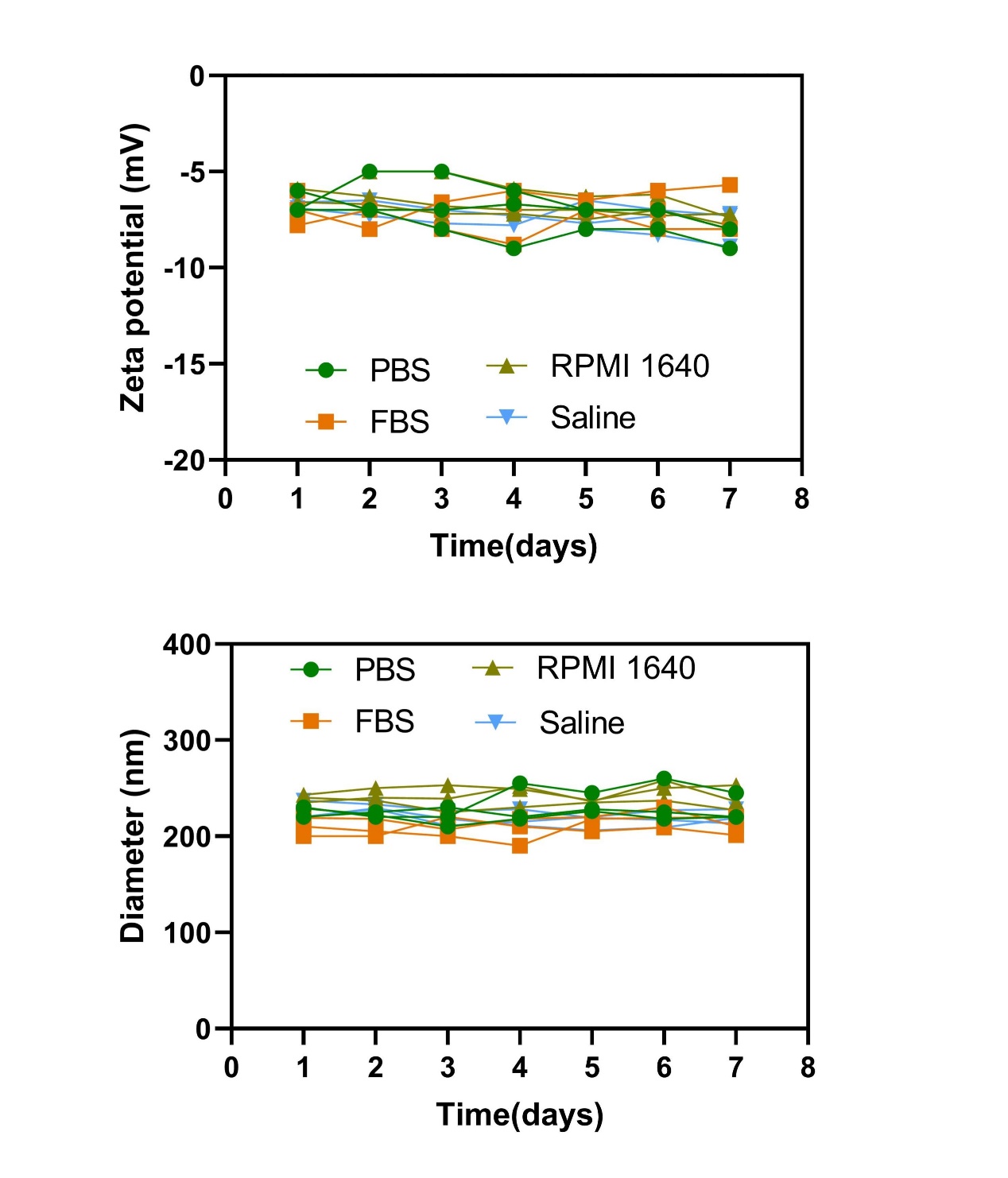


**Figure S13.** A) Zeta potential of the DNMF/PLGA NPs within 7 days. B) Size distribution of the DNMF/PLGA NPs within 7 days. Data are presented as the means ±SD. ANOVA with Dunnett's post-hoc test.


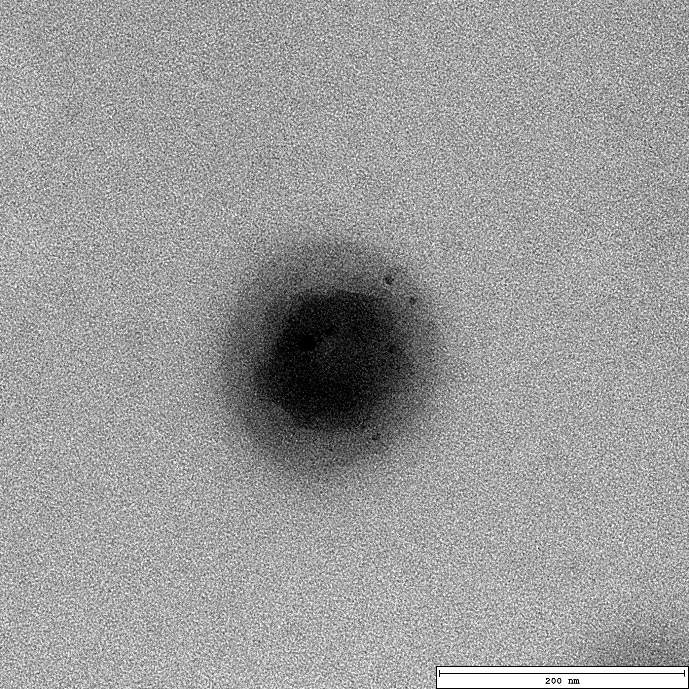


**Figure S14.** TEM image of the DNMF/PLGA NPs.

**
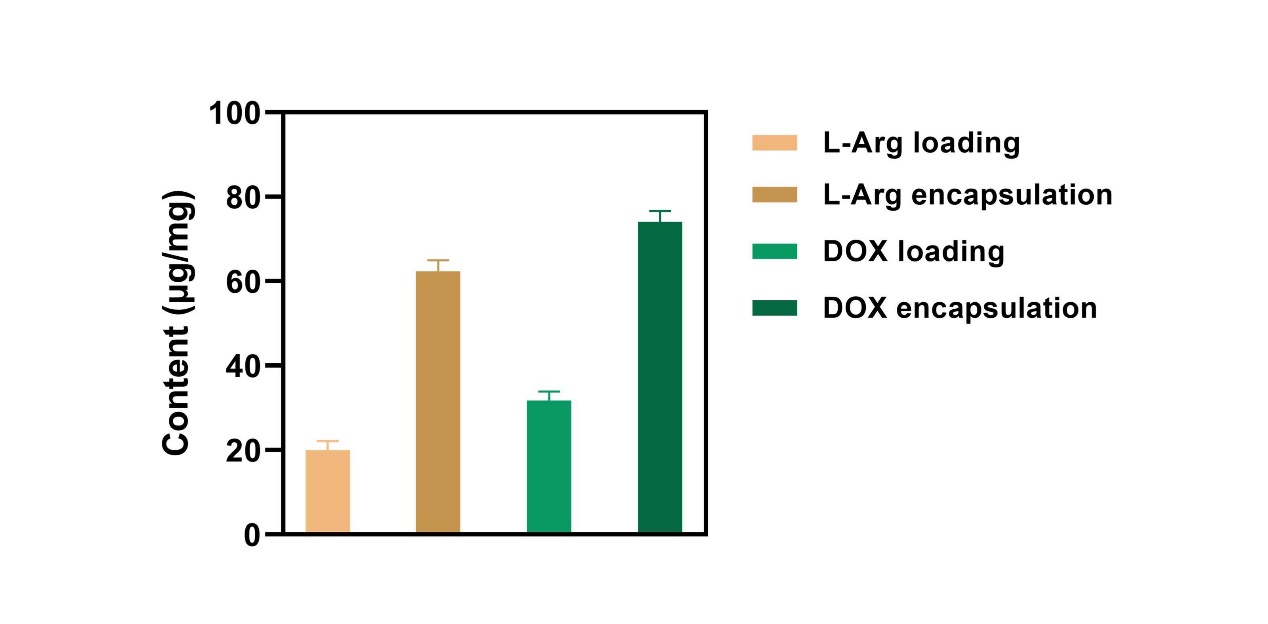
**

**Figure S15.** The amount of DOX loading and encapsulation, the amount of L-Arg loading and encapsulation (n = 3).


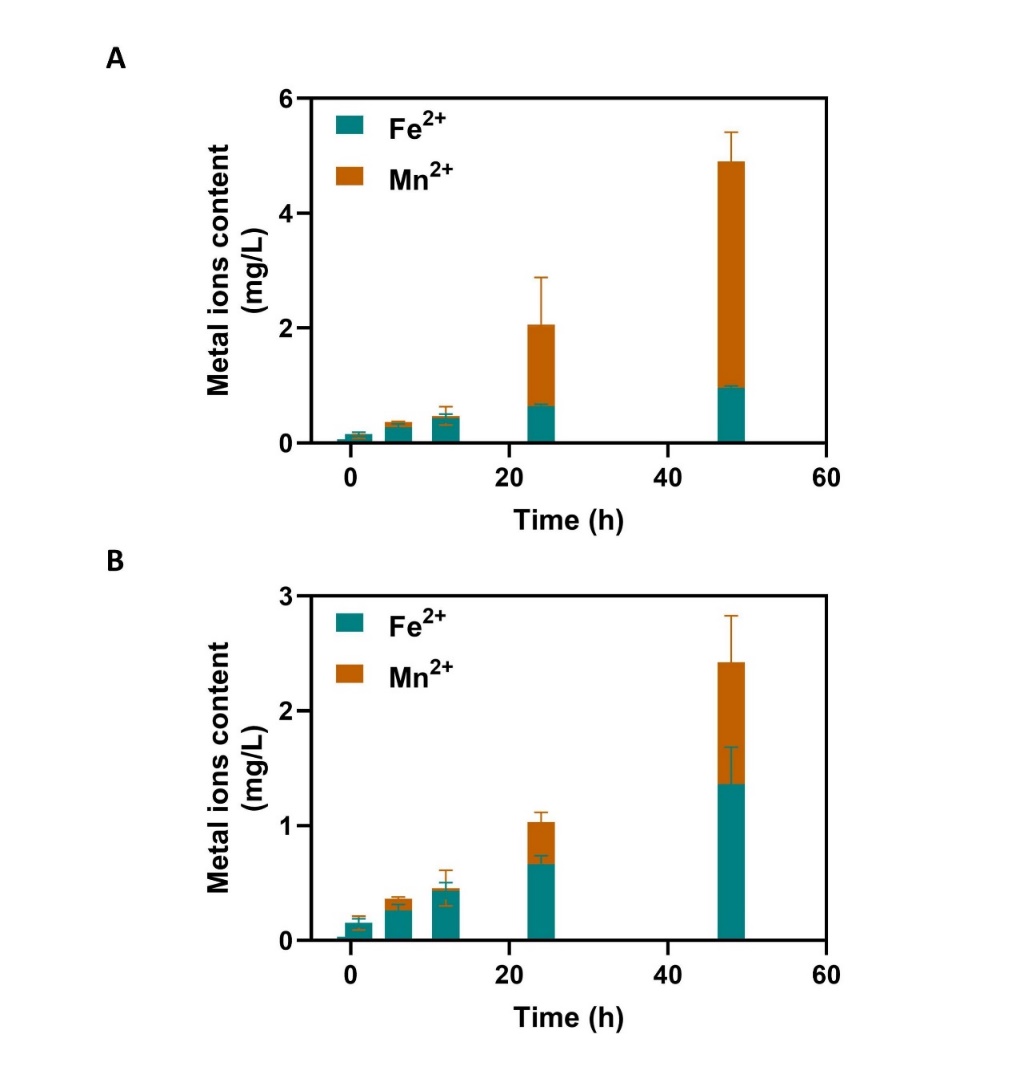


**Figure S16.** A) Following 808-nm laser irradiation for 5 min, Mn^2+^ and Fe^2+^release in physiological state was observed at time point of 0h, 1h, 6h, 12h, 24h, 48h. B) Without laser irradiation.


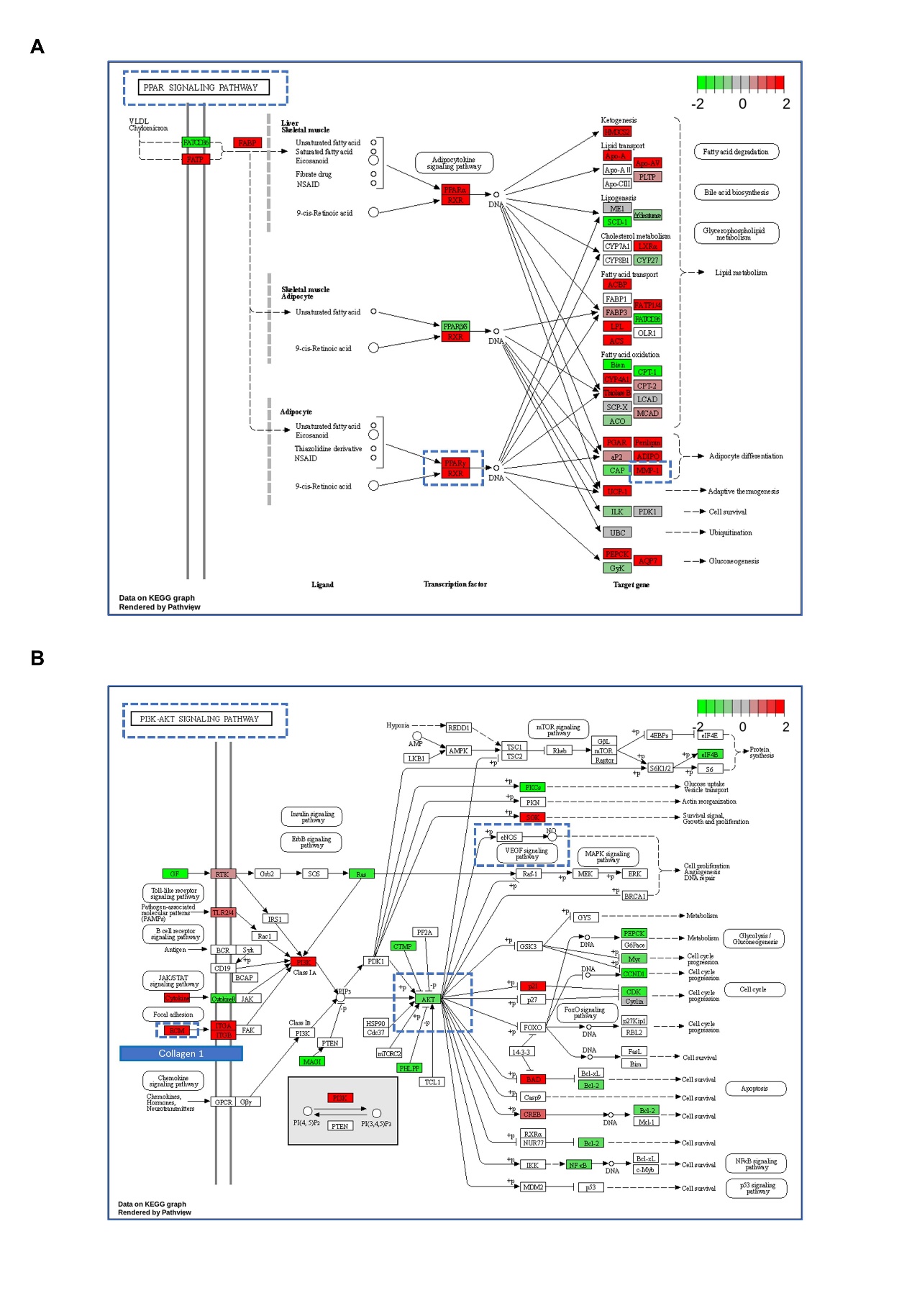


**Figure S17.** Mechanistic analysis of MMPs activity and collagen I reduction. A) MMP-1 target gene data on KEGG graph rendered by pathview. B) Collagen I related gene data on KEGG graph rendered by pathview. A warm red color indicates significantly upregulated genes, while a cold green color represents remarkably downregulated genes.

**Table S1.** The comparison of NO-reactor nanoparticles

| NP name | NO donor | Efficiency of NO production | Main mechanism | Reference |
| --- | --- | --- | --- | --- |
| DNMF/PLGA | L-Arg | 68% | MMPs inhibition, collagen degradation, tumor inhibition |  |
| DN@MSN | S-nitroso thiols | 83% | MMPs inhibition, collagen degradation | [28] |
| DM1-NO/PLGA | DM1-NO | 86% | Tumor inhibition | [29] |
| Ag_2_S@BSA | Tert-Butyl nitrite | Not available | Tumor radiosensitization | [27] |
